# Effects of Molecular Aggregation on Dynamic Third-Order Nonlinear Optical Responses: Oligo(thiophene-benzothiadiazole) as a Case Study

**DOI:** 10.1021/acs.jctc.6c00268

**Published:** 2026-04-21

**Authors:** Karan Ahmadzadeh, Robert Zaleśny, Xin Li, Zilvinas Rinkevicius, Wei Hu, Patrick Norman

**Affiliations:** † Hefei National Research Center for Physical Sciences at the Microscale, 12652 University of Science and Technology of China, Hefei, Anhui 230026, China; ‡ Faculty of Chemistry, 49567 Wrocław University of Science and Technology, Wyb. Wyspiánskiego 27, Wrocław PL-50370, Poland; § PDC Center for High Performance Computing, 7655 KTH Royal Institute of Technology, Stockholm SE-100 44, Sweden; ∥ Division of Theoretical Chemistry and Biology, School of Engineering Sciences in Chemistry, Biotechnology and Health, 7655 KTH Royal Institute of Technology, Stockholm SE-100 44, Sweden; ⊥ State Key Laboratory of Precision and Intelligent Chemistry, 12652 University of Science and Technology of China, Hefei, Anhui 230026, China

## Abstract

Nonlinear optical properties of molecular materials are governed by aggregation and cooperative effects in the condensed phase, requiring first-principles simulations on large molecular assemblies to achieve a realistic description of experiment. While modern electronic-structure theory implementations mitigate key computational challenges through low-memory two-electron integral engines and multifrequency response solvers, practical calculations of third-order nonlinear optical response in extended systems remain prohibitively expensive. The dominant bottleneck arises because conventional approaches require evaluating many independent components of the rank-four hyperpolarizability tensor, each of which is costly, making orientational averaging prohibitively expensive. Here, we introduce an analytic tensor-averaged formulation of cubic response theory within time-dependent Kohn–Sham density functional theory that removes this spatial bottleneck. By performing the orientational average directly at the level of perturbed densities and transformed Fock matrices, the full Cartesian second-order hyperpolarizability tensor is never constructed. Instead, the isotropic second-order hyperpolarizability is obtained directly. Exploiting the linearity of the Fock-matrix construction, this approach eliminates redundant spatial components and reduces the number of required exchange–correlation kernel integrations in the electronic quartic- and cubic-Hessian contractions by 72 and 90%, respectively. The resulting methodology enables cubic-response simulations of third-harmonic generation in large molecular aggregates with exchange–correlation functionals spanning multiple rungs of Jacob’s ladder. Applications to oligo­(thiophene-benzothiadiazole) (OTBP) clusters reveal pronounced aggregation-induced dampening of the third-harmonic response and demonstrate scalability to systems with nearly 5800 contracted basis functions. The present framework provides a quantitative tool for disentangling intrinsic molecular effects from supramolecular and morphological contributions, and for assessing the potential for further performance gains through crystal-structure engineering. In this way, theory can directly guide experimental efforts by identifying packing motifs that maximize cooperative enhancement and those that are detrimental. By resolving the missing spatial-domain optimization in the cubic response theory, the present tensor-averaged formulation shifts the dominant computational cost back to the frequency-dependent response solver, enabling routine material-level modeling of cooperative third-order nonlinear optical phenomena.

## Graphical abstract

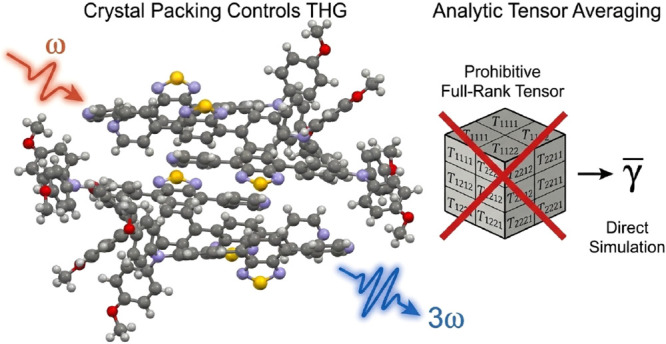

## 1 Introduction

Nonlinear optical (NLO) materials are central to modern photonics, enabling applications such as ultrafast signal processing,^1^ optical frequency conversion,^2^ and label-free imaging. Among third-order nonlinear processes, third-harmonic generation (THG) is particularly attractive because it is allowed in all materials regardless of symmetry and provides intrinsic structural contrast in biological media.^3,4^ THG microscopy^5^ combines deep tissue penetration with submicrometer spatial resolution, allowing direct visualization of cellular and subcellular interfaces, including membranes, lipid droplets, and myelin sheaths, without the use of fluorescent labels.^6,7^ Despite these advantages, THG signals in soft and biological media are inherently weak due to small third-order susceptibilities and a low refractive-index contrast. This limitation has motivated the development of engineered molecular systems with enhanced nonlinear responses for use as THG contrast agents.

Strategies for enhancing the nonlinear optical response fall into two main categories based on the electronic and morphological contributions to the third-order susceptibility. The first category is electronic enhancement, in which χ^(3)^ is increased by modifying the chemical structure of the constituent molecules, thereby altering their electronic structure and enhancing the intrinsic molecular hyperpolarizability γ. This can be achieved through molecular design strategies such as donor–acceptor architectures, extended π-conjugation, improved backbone planarity, and increased charge-transfer character. In addition, γ can be further enhanced through electronic resonance, where the excitation frequency is tuned near a molecular electronic transition, leading to a frequency-dependent amplification of the nonlinear polarization.^8,9^ The second category is morphology-driven enhancement. In this case, the molecular hyperpolarizability γ remains unchanged, but the local electromagnetic field interacting with the molecules is increased. High-refractive-index materials, dielectric interfaces, and nanostructures that support strong optical scattering or confinement reshape and concentrate the local field. This local field enhancement increases the effective macroscopic χ^(3)^ through refractive-index contrast and field-focusing effects.

The theoretical design of third-order nonlinear optical materials begins with the hyperpolarizability γ and commonly involves a two-step process involving molecular and supramolecular design.^10^ The molecular step focuses on optimizing the electronic structure of individual chromophores, typically donor–acceptor π-conjugated systems, to achieve large intrinsic hyperpolarizabilities.^11–13^ The supramolecular step concerns the organization of these chromophores into aggregates, crystals, or films, where intermolecular interactions can cooperatively enhance or suppress the macroscopic nonlinear response. Accurate prediction of NLO properties, therefore, requires descriptions that capture both intrinsic molecular polarizability and collective interactions in the condensed phase.

A central difficulty in theoretical investigations of the nonlinear optical theory lies in extrapolating from microscopic molecular hyperpolarizabilities to macroscopic bulk susceptibilities. While molecular-scale design principles are now well established, predictive supramolecular modeling remains far less developed. Early efforts to relate molecular and bulk nonlinear responses relied primarily on the oriented-gas approximation,^14–16^ which treats the macroscopic susceptibility as the tensorial sum of the responses of noninteracting, oriented monomers, modified only by local-field factors. However, numerous theoretical and experimental studies have since shown that this additivity-based approximation breaks down, as cooperative intermolecular effects can strongly enhance or suppress the overall NLO response.^17–23^

In molecular aggregates, intermolecular interactions give rise to two key cooperative phenomena. Excitonic coupling,^24,25^ arises from electronic interactions between the transition densities of neighboring chromophores, leading to delocalization of excited states and collective optical responses. Polarization effects, in turn, originate from mutual electrostatic induction, whereby the local electric field produced by one molecule distorts the charge distribution of its neighbors. These effects can substantially enhance or suppress the overall NLO response, making it nonadditive with respect to individual molecular contributions.^26–28^

To account for the effects mentioned above, a natural extension of the oriented-gas model has been to replace the isolated monomer with small clusters of explicitly interacting chromophores representing the contents of the unit cell. In this cluster approach, dimers, trimers, or local crystal fragments are treated within a single quantum-mechanical calculation, enabling intermolecular polarization, charge redistribution, and some excitonic coupling to be incorporated directly into the evaluation of the nonlinear response. Several early studies employing this strategy have shown that including intermolecular interactions at the cluster level yields improved agreement between calculated and experimental macroscopic susceptibilities.^17,29–32^

To date, most theoretical investigations of aggregation effects on nonlinear optical properties have been limited to the static or far from resonance frequencies of small molecular aggregates, where excitonic and polarization couplings have been treated explicitly at the DFT or MP2 levels of theory.^10,10,33–36^ Finite-field and coupled perturbed DFT methods have also been applied to crystalline systems under periodic boundary conditions,^37–40^ which have provided valuable benchmark data, yet still offer limited microscopic insight into the mechanisms of cooperative enhancement.

To overcome these size limitations, simplified excitonic models, most notably the two-state model (TSM) and its molecular-exciton extensions, have been employed to approximate intermolecular coupling effects in large chromophore assemblies.^25^ While these approaches achieve remarkable computational efficiency and can handle disordered aggregates containing thousands of chromophores, they rely on parametrization from TDDFT and restrict the electronic manifold to only a few states, thereby sacrificing first-principles accuracy and transferability.

Besides excitonic and few-state models, simplified response methods such as sTDA,^41^ sTDDFT,^42^ and XsTDDFT,^43^ have also shown promise for low-cost calculations of optical response properties and hyperpolarizabilities in large systems.^44,45^ These approaches achieve their efficiency by introducing additional approximations in the treatment of the electron–electron interaction, thereby avoiding the full cost of conventional two-electron integral evaluations.

When the perturbation varies in time, the system no longer possesses a well-defined energy, and dynamic response properties must instead be described within explicitly time-dependent formalisms such as the Ehrenfest^46^ or the quasi-energy formulations.^47,48^ Consequently, static finite-field methods are inherently incapable of describing frequency-dependent and resonance-enhanced nonlinear optical processes.

Although rigorous response-theory formalisms for frequency-dependent optical properties are well-established in principle, their practical application within DFT remains severely hampered by unfavorable computational scaling and by the limited accuracy of present exchange-correlation functionals, limitations that become especially acute in aggregated and multichromophoric systems. As a result, quantitative modeling of nonlinear optical processes in extended molecular systems demands both more efficient electronic structure algorithms and the development of new functionals capable of accurately treating cubic response.

Accurate modeling of resonance-enhanced NLO processes poses an additional fundamental challenge. Traditional time-dependent perturbation theory becomes numerically unstable near electronic resonances because excited-state relaxation is not explicitly included in the Hamiltonian. As a result, conventional response expressions diverge or become ill-conditioned precisely in the spectral regions most relevant for resonance-enhanced nonlinear optics. To overcome these limitations, the complex polarization propagator (CPP), or damped-response, formalism was developed, introducing finite lifetimes of electronically excited states directly into the response equations.^49^ This approach yields smooth, physically meaningful frequency-dependent susceptibilities across both nonresonant and resonant regimes. Subsequent developments have extended the CPP framework to higher-order nonlinear responses and to Kohn–Sham DFT, establishing the resonant-convergent damped-response theory.^50–54^

Accurate prediction of molecular polarizabilities remains a key challenge in DFT. Most approximate exchange–correlation functionals, however, tend to produce electron densities that are too soft, that is, overly delocalized and excessively responsive to external electric fields, leading to systematic overestimation of polarizabilities.^55^ Accurate predictions of polarizabilites and hyperpolarizabilites often require a proper description of electron correlation. Coupled-cluster methods (CC), though computationally more demanding, offer highly reliable results because they include systematic treatments of electron correlation. In particular, for static polarizabilites, the CCSD­(T) model, which includes single and double excitations with a perturbative treatment of triple excitations, is often considered the “gold standard” for quantum chemistry because of its outstanding accuracy; for dynamic polarizabilites, it is the CC3 method. Wu et al^56^ conducted a comprehensive benchmark of DFT functionals and demonstrated that functionals such as M06-2X and M11 provide some of the most reliable results when compared to high-level CCSD­(T) reference data. A more recent benchmark study employing larger CCSD­(T)/CBS-quality data sets and including double-hybrid functionals has since shown that while hybrid functionals typically yield mean relative errors of 4–5%, double hybrids achieve improved accuracy in the range of 2.5–3.8%. However, it is important to emphasize that performance for one response property does not automatically transfer to another. In particular, a functional that performs well for the first hyperpolarizability β may still be less reliable for the second hyperpolarizability γ, and vice versa. In particular, double hybrid functionals such as XYGJ-OS and XYG3 deliver near-CCSD­(T) performance, whereas advanced meta-GGAs like mBEEF and MVS provide hybrid-level accuracy at significantly lower computational cost.^55^ The use of double hybrid functionals for the calculation of dynamic polarizabilites and hyperpolarizabilites is, however, problematic as it may contain pole instabilities, such as in the Mo̷ller–Plesset-based response theory.^57^ While the polarizability corresponds to the first-order (linear) response of the electron density to an external electric field, many optically relevant quantities, such as hyperpolarizabilities and two-photon absorption (2PA) cross-sections, arise from higher-order (nonlinear) response functions. Extending the benchmarking of density functionals to these nonlinear optical properties, recent studies have demonstrated that TDDFT provides a strong Pearson correlation for 2PA transition strengths and cross-sections relative to high-level RI-CC2 reference data.^58–61^

Despite these theoretical advances, the quantitative simulation of frequency-dependent third-order nonlinear-optical properties in large molecular aggregates remains computationally prohibitive. Modern response theory implementations already mitigate several major cost drivers through two key algorithmic developments. First, lightweight two-electron integral engines with minimal memory footprint enable the efficient construction of many auxiliary Fock matrices from a single batched evaluation of electron repulsion integrals.^62^ Second, subspace multifrequency response solvers permit the simultaneous treatment of multiple excitation frequencies within a common iterative subspace, allowing entire spectral windows to be converged at a computational cost only marginally exceeding that of a single frequency.^53,54^

However, even within this highly optimized framework, large-scale nonlinear-response calculations remain fundamentally limited by a distinct and largely unaddressed spatial domain bottleneck. In the conventional tensor-component formulation, the evaluation of isotropic third-order response properties requires the explicit construction of all Cartesian components of the rank-four hyperpolarizability tensor. Each of the Cartesian tensor elements demand independent cubic- and quartic-Hessian contractions, which in turn require the repeated construction of large numbers of transformed Fock matrices. Although the underlying two-electron integrals can in principle be reused, the rapid growth of the auxiliary Fock matrices with basis set size precludes their simultaneous construction and storage in memory. As a consequence, the Fock matrices must be generated in successive memory-limited batches, and the repeated linear transformations within each batch become the dominant sources of computational and memory overhead in large-scale cubic-response calculations.

Importantly, this bottleneck is not associated with the electronic structure of the chromophores or with the solution of the response equations themselves but rather with the explicit treatment of Cartesian spatial tensor components required to perform orientational averaging. Removing this redundancy at the level of the transformed densities and Fock matrices therefore represents a fundamentally different route to algorithmic acceleration than that of existing frequency-domain optimizations. The tensor-averaged formulation introduced in this work directly targets this spatial-domain bottleneck by applying the orientational average analytically prior to the electronic Hessian contractions, thereby eliminating redundant Cartesian tensor elements and shifting the dominant computational cost back to the response solvers themselves.

The goal of this work is to investigate resonance enhancement, excitonic coupling, and the scaling of the third-order response with aggregate size. To that end, we analyze oligo­(thiophene-benzothiadiazole) (OTBP) aggregates. OTBP nanocrystals were recently developed as an ultraefficient organic contrast agent for backward THG-based angiographic imaging.^63^ OTBP was specifically designed to maximize both the molecular and morphological contributions to the nonlinear response. Furthermore, resonant enhancement was used to enhance the intrinsic molecular nonlinearity, while the optimized crystalline nanostructure supports Mie-type photonic resonances that amplify the local electromagnetic field. The synergy between these effects enables high-contrast, deep-tissue vascular imaging at sub-10 mW excitation powers.^63^

To achieve this goal, we present an efficient implementation of cubic response theory combined with the time-dependent Kohn–Sham density functional theory. A tensor-averaging algorithm was introduced to construct damped isotropic cubic response quantities directly, eliminating redundant tensor components and reducing computational cost by nearly an order of magnitude. These developments make it possible, for the first time, to perform analytic TDDFT-level cubic response calculations on molecular aggregates, capturing excitonic and polarization effects from first principles. The developed methodology enables efficient computational studies of third-order NLO effects using exchange-correlation functionals from various rungs of Jacob’s ladder, including meta-GGA functionals. More broadly, this framework establishes a practical route for predictive, material-level simulations of nonlinear optical phenomena, linking electronic-structure accuracy with mesoscale aggregation effects. It provides a foundation for the computational design of next-generation photonic and bioimaging materials with tailored third-order optical properties.

The remainder of this paper is organized as follows. In Section 2, we briefly review the theoretical foundations of third-harmonic generation and the quasi-energy response framework, and introduce the orbital-rotation parametrization underlying the time-dependent Kohn–Sham formalism. We then present the tensor-component formulation of cubic response theory and derive an analytic tensor-averaged approach that eliminates the explicit construction of Cartesian hyperpolarizability components, thereby removing the dominant spatial-domain bottleneck. Section 3 applies the developed methodology to oligo­(thiophene-benzothiadiazole) (OTBP) systems, beginning with an analysis of resonance-enhanced third-harmonic generation and the underlying multiphoton mechanisms in isolated chromophores, followed by a detailed investigation of aggregation-induced modulation of the nonlinear response in experimentally relevant packing motifs. The computational efficiency and scaling behavior of the tensor-averaged formulation are also assessed in this section.

## 2 Theoretical Framework and Implementation

### 2.1 Third-Harmonic Generation and Molecular Second Hyperpolarizabilities

Third-harmonic generation (THG) is driven by the third-order macroscopic polarization, which acts as a nonlinear source term in Maxwell’s wave equation,

$$\nabla^{2}F(3\omega )-\frac{n^{2}(3\omega )}{c^{2}}\frac{\partial^{2}F(3\omega )}{\partial t^{2}}=\frac{1}{\varepsilon_{0}c^{2}}\frac{\partial^{2}P^{\left(3\right)}(3\omega )}{\partial t^{2}}$$ 1

where ***F*** (3ω) is the generated third-harmonic field, *n*(3ω) is the refractive index at the harmonic frequency, and ***P*** ^(3)^(3ω) is the third-order nonlinear polarization, which represents the third-order correction to the dipole moment per unit volume. In nonlinear optics, the *n*th-order contribution to the macroscopic polarization at the output frequency ω_σ_ = −(ω_1_ + ... + ω_*n*_) is written as^64^

$$P_{I_{n+1}}^{\left(n\right)}(\omega_{\sigma })=\chi_{I_{n+1}I_{1}...I_{n}}^{(n)\mathrm{eff}}(-\omega_{\sigma };\omega_{1},...,\omega_{n})F_{I_{1}}(\omega_{1})...F_{I_{n}}(\omega_{n})$$ 2

where χ^(*n*)eff^ is the effective macroscopic susceptibility, including both orientational averaging and local-field effects. For third–order nonlinear processes such as third-harmonic generation (THG), the oriented-gas model provides a microscopic description of the macroscopic third-order susceptibility by treating the response as the sum of the individual molecular contributions within the crystallographic unit cell. Under the assumption that intermolecular interactions are weak relative to the intrinsic intramolecular response, the susceptibility may be written as^14^

$$\chi_{IJKL}^{(3),\mathrm{eff}}(-\omega_{\sigma };\omega_{1},\omega_{2},\omega_{3})=\frac{1}{V}f_{I}(\omega_{\sigma })f_{J}(\omega_{1})f_{K}(\omega_{2})f_{L}(\omega_{3})\sum_{s=1}^{N_{\mathrm{mol}}}\sum_{i,j,k,l}C_{Ii}^{s}C_{Jj}^{s}C_{Kk}^{s}C_{Ll}^{s}\gamma_{ijkl}^{\left(s\right)}(-\omega_{\sigma };\omega_{1},\omega_{2},\omega_{3})$$ 3

where *V* is the unit-cell volume, *f*_*I*_(ω) are local-field factors, *C*_*Ii*_^*s*^ are direction cosines relating the molecular and crystal frames, and γ_*ijkl*_^(*s*)^ denotes the molecular third–order hyperpolarizability tensor. In many molecular solids, however, the electronic response is substantially modified by intermolecular effects such as hydrogen bonding, charge transfer, and mutual polarization, and the additivity assumption implicit in eq 3 is no longer representative for the real material. A natural extension to the oriented gas model is to replace the explicit molecular sum by an effective hyperpolarizability for the entire unit cell, and, when an isotropic and orientationally averaged response is required, to combine this substitution with the conventional rotational average of the cluster tensor. These considerations lead to the compact expression

$$\chi_{IJKL}^{(3),\mathrm{eff}}=\frac{1}{V}f_{I}(\omega_{\sigma })f_{J}(\omega_{1})f_{K}(\omega_{2})f_{L}(\omega_{3}){\mathrm{\gamma ̅}}_{\mathrm{cell}}(-\omega_{\sigma };\omega_{1},\omega_{2},\omega_{3})$$ 4

in which γ̅_cell_ denotes the effective (cluster) hyperpolarizability of the unit cell or, for isotropic media, its orientationally averaged counterpart. The latter is given by the familiar rotational average,^65^

$$\mathrm{\gamma ̅}(-\omega_{\sigma };\omega ,\omega ,\omega )=\frac{1}{15}\sum_{\alpha ,\beta =x,y,z}(\gamma_{\alpha \alpha \beta \beta }+\gamma_{\alpha \beta \beta \alpha }+\gamma_{\alpha \beta \alpha \beta })$$ 5

so that the microscopic (electronic) response enters solely through *γ̅*_cell_, while the morphological properties of the medium remain entirely encoded in the local-field factors. The molecular polarizabilities and hyperpolarizabilities arise naturally from the perturbative expansion of the time-dependent electric-dipole moment in powers of the external electric field. Writing the time-dependent dipole moment in terms of its Fourier components,

$$\mu (t)=\sum_{a}(\mu (\omega_{a})e^{-i\omega_{a}t}+\mu (-\omega_{a})e^{i\omega_{a}t})$$ 6

Fourier amplitude at output frequency ω_σ_ admits the expansion

$$\mu_{\alpha }(\omega_{\sigma })=\mu_{\alpha }^{\omega_{\sigma }}+\sum_{\beta }\sum_{\omega_{a}}\alpha_{\alpha \beta }(-\omega_{\sigma };\omega_{a})F_{\beta }^{\omega_{a}}+\sum_{\beta ,\gamma }\sum_{\omega_{a},\omega_{b}}\frac{1}{2}\beta_{\alpha \beta \gamma }(-\omega_{\sigma };\omega_{a},\omega_{b})F_{\beta }^{\omega_{a}}F_{\gamma }^{\omega_{b}}+\sum_{\beta ,\gamma ,\delta }\sum_{\omega_{a},\omega_{b},\omega_{c}}\frac{1}{6}\gamma_{\alpha \beta \gamma \delta }(-\omega_{\sigma };\omega_{a},\omega_{b},\omega_{c})F_{\beta }^{\omega_{a}}F_{\gamma }^{\omega_{b}}F_{\delta }^{\omega_{c}}+...$$ 7

which defines the molecular polarizability α, first hyperpolarizability β, and second hyperpolarizability γ as^66^

$$\begin{array}{lll} \alpha_{\alpha \beta }=\frac{d\mu_{\alpha }}{dF_{\beta }}{|}_{\mathrm{F̅}=0}, & \beta_{\alpha \beta \gamma }=\frac{d^{2}\mu_{\alpha }}{dF_{\beta }dF_{\gamma }}{|}_{\mathrm{F̅}=0}, & \gamma_{\alpha \beta \gamma \delta }=\frac{d^{3}\mu_{\alpha }}{dF_{\beta }dF_{\gamma }dF_{\delta }}{|}_{\mathrm{F̅}=0} \end{array}$$ 8

### 2.2 Quasi-Energy Response Framework for Molecular Hyperpolarizabilities

Within the quasi-energy formulation, the equation governing the responses of the wave function parameters κ to the perturbation is derived from the time-dependent variational principle for the time-averaged quasi-energy.^47^ The field expansion for this is given by the expression as

$$\frac{d}{dF_{\beta }^{\omega_{1}}}\left(\frac{\partial Q_{T}}{\partial \kappa_{n}^{\omega_{\sigma }}}\right){|}_{\begin{array}{c} F=0 \end{array}}F_{\beta }^{\omega_{1}}+\frac{d^{2}}{dF_{\beta }^{\omega_{1}}dF_{\gamma }^{\omega_{2}}}\left(\frac{\partial Q_{T}}{\partial \kappa_{n}^{\omega_{\sigma }}}\right){|}_{\begin{array}{c} F=0 \end{array}}F_{\beta }^{\omega_{1}}F_{\gamma }^{\omega_{2}}+...=0$$ 9

where the time-averaged quasi-energy is defined in terms of the time-independent Hamiltonian *Ĥ*_0_, and the time-dependent perturbation operator *V* ^(*t*)­

$$Q_{T}=\frac{1}{T}\int_{-T/2}^{T/2}\langle \Psi (t)|\left({Ĥ}_{0}+\mathrm{V̂}(t)-i\frac{\partial }{\partial t}\right)|\Psi (t)\rangle dt$$ 10

One can show that the quasi-energy satisfies the time-dependent Hellman–Feynman theorem,

$$\frac{dQ_{T}}{dF_{\alpha }^{\omega_{1}}}=-\frac{1}{T}\int_{-T/2}^{T/2}\langle 0̃|{\mathrm{\mu ̂}}_{\alpha }|0̃\rangle e^{-i\omega_{1}t}dt$$ 11

In the presence of the field, the quasi-energy is written as a power series expansion of the Fourier amplitudes of the field

$$Q_{T}(F)=\sum_{\omega_{k}}\sum_{\alpha }\frac{dQ_{T}}{dF_{\alpha }^{\omega_{k}}}{|}_{F=0}F_{\alpha }^{\omega_{k}}+\frac{1}{2!}\sum_{\omega_{k},\omega_{l}}\sum_{\alpha ,\beta }\frac{d^{2}Q_{T}}{dF_{\alpha }^{\omega_{k}}dF_{\beta }^{\omega_{l}}}{|}_{F=0}F_{\alpha }^{\omega_{k}}F_{\beta }^{\omega_{l}}+\frac{1}{3!}\sum_{\omega_{k},\omega_{l},\omega_{m}}\sum_{\alpha ,\beta ,\gamma }\frac{d^{3}Q_{T}}{dF_{\alpha }^{\omega_{k}}dF_{\beta }^{\omega_{l}}dF_{\gamma }^{\omega_{m}}}{|}_{F=0}F_{\alpha }^{\omega_{k}}F_{\beta }^{\omega_{l}}F_{\gamma }^{\omega_{m}}+...$$ 12

Using the time-dependent Hellman–Feynman theorem of eq 11, it can be shown that the quasi-energy derivatives correspond to the Fourier coefficients of the polarizability and hyperpolarizabilities,^48^

ααβ(−ωσ;ωk)=−⟨⟨μ̂α;μ̂β⟩⟩ωk=−d2QTdFα−ωσdFβωk|F=0βαβγ(−ωσ;ωk,ωl)=⟨⟨μ̂α;μ̂β,μ̂γ⟩⟩ωk,ωl=d3QTdFα−ωσdFβωkdFγωl|F=0γαβγδ(−ωσ;ωk,ωl,ωm)=−⟨⟨μ̂α;μ̂β,μ̂γ,μ̂δ⟩⟩ωk,ωl,ωm=−d4QTdFα−ωσdFβωkdFγωldFδωm|F=0 13

### 2.3 Orbital-Rotation Parameterization of the Kohn–Sham Reference State

The time evolution of the Kohn–Sham determinant is parametrized in terms of the time-dependent orbital rotations

$$\left|\Psi (t)\rangle =e^{-\mathrm{\kappa ̂}(t)}|0\right\rangle$$ 14

The anti-Hermitian orbital-rotation operator *κ̂* can be written as an inner product

$$\begin{array}{r} \mathrm{\kappa ̂}\left(t\right)=\left(\begin{array}{ll} {\mathrm{q̂}}_{n}^{†} & -{\mathrm{q̂}}_{n} \end{array}\right)\left(\begin{array}{l} \kappa_{n}\left(t\right)\\ \kappa_{n}^{*}\left(t\right) \end{array}\right)=\underset{a,i}{\sum }\left(\kappa_{ai}\left(t\right){â}_{a}^{†}{â}_{i}-\kappa_{ai}^{*}\left(t\right){â}_{i}^{†}{â}_{a}\right) \end{array}$$ 15

where the excitation and de-excitation operators are given by

$${\mathrm{q̂}}_{n}^{†}={â}_{a}^{†}{â}_{i},{\mathrm{q̂}}_{n}={â}_{i}^{†}{â}_{a}$$ 16

with indices *i*, *j*,..., *a*, *b*,..., and *p*, *q*,... we denote occupied, unoccupied, and general spin orbitals or spatial orbitals when the operators are accompanied by a spin index, respectively. We also introduce compound indices, *n*, *m*,..., to denote pairs of orbital indices {*a*, *i*}. Furthermore, we use Greek letters for Cartesian coordinates in the molecular frame of reference. Using eq 15, we define the state-rotation parameter vector in two blocks as

$$\kappa =\left(\begin{array}{l} \kappa_{n}\\ \kappa_{n}^{*} \end{array}\right)$$ 17

Using the Baker–Campbell–Hausdorff expansion, one can expand the density in terms of the orbital-rotation parameters as,^67^

$$\rho (r,t)=\langle 0|e^{\mathrm{\kappa ̂}(t)}\mathrm{\rho ̂}(r)e^{-\mathrm{\kappa ̂}(t)}|0\rangle =\langle 0|\mathrm{\rho ̂}(r)|0\rangle +\sum_{n}\langle 0|[\kappa_{r}(t){\mathrm{q̂}}_{n}^{†}-\kappa_{n}^{*}(t){\mathrm{q̂}}_{n},\mathrm{\rho ̂}(r)]|0\rangle +\frac{1}{2!}\sum_{n,m}\langle 0|[\kappa_{r}(t){\mathrm{q̂}}_{n}^{†}-\kappa_{n}^{*}(t){\mathrm{q̂}}_{n},[\kappa_{m}(t){\mathrm{q̂}}_{m}^{†}-\kappa_{m}^{*}(t){\mathrm{q̂}}_{m},\mathrm{\rho ̂}(r)]]|0\rangle +...$$ 18

For a general approximate functional employed in this work, the XC functional depends on the spin densities ρ_σ_(**r**), their gradients ∇ρ_σ_(**r**), their laplacian ∇^2^ρ_σ_(**r**), and the orbital kinetic energy densities τ_σ_(**r**), and can be written in the general form

$$E_{\mathrm{xc}}=\int e_{\mathrm{xc}}(\rho_{↑}(r),\rho_{↓}(r),\nabla \rho_{↑}(r),\nabla \rho_{↓}(r),\nabla^{2}\rho_{↑}(r),\nabla^{2}\rho_{↓}(r),\tau_{↑}(r),\;\tau_{↓}(r))d^{3}r$$ 19

Here, the orbital kinetic energy density is given by

$$\tau_{\sigma }(r)=\sum_{i=1}^{N_{\sigma }}|\nabla \psi_{i\sigma }(r){|}^{2}$$ 20

Since τ_σ_(**r**) depends explicitly on the Kohn–Sham orbitals, the exchange–correlation functional is not an explicit functional of the density. The inclusion of the kinetic energy density, however, introduces two practical challenges. First, τ_σ_(**r**) is not an explicit functional of the electron density, which complicates the evaluation of functional derivatives with respect to the density. One formal approach to address this issue is the optimized effective potential (OEP) method,^68,69^ which provides an exact treatment but is computationally demanding. Alternatively, τ_σ_(**r**) can be treated as an explicit variable, allowing the evaluation of functional derivatives with respect to the orbitals rather than the density. This practical solution, often referred to as the orbital-dependent density functional method (ODDM), underlies most modern implementations of meta-GGA functionals and enables their extension to time-dependent DFT (TDDFT) formulations.^70,71^ . Another issue is that the conventional kinetic energy density, τ_σ_(**r**), is not gauge invariant. A well-established approach to restore gauge invariance is to construct a generalized kinetic energy density, *τ̂*_σ_(**r**), by explicitly incorporating the paramagnetic current density. This modification leads to current-dependent meta-GGA (cMGGA) functionals that yield gauge-consistent exchange-correlation potentials and response properties.^72–76^ In this work, we adopt the orbital-dependent density functional method (ODDM), in which τ_σ_ is treated as an explicit basic variable and functional. For a generic functional of the form of eq 19, the relevant operators are therefore

$$\begin{array}{c} {\mathrm{\rho ̂}}_{\sigma }=\sum_{pq}\psi_{p;\sigma }^{†}\psi_{q;\sigma }{â}_{p;\sigma }^{†}{â}_{q;\sigma }\\ \nabla {\mathrm{\rho ̂}}_{\sigma }=\sum_{pq}\nabla (\psi_{p;\sigma }^{†}\psi_{q;\sigma }){â}_{p;\sigma }^{†}{â}_{q;\sigma }\\ \nabla^{2}{\mathrm{\rho ̂}}_{\sigma }=\sum_{pq}\nabla^{2}(\psi_{p;\sigma }^{†}\psi_{q;\sigma }){â}_{p;\sigma }^{†}{â}_{q;\sigma }\\ {\mathrm{\tau ̂}}_{\sigma }=\sum_{pq}(\nabla \psi_{p;\sigma }^{†}\cdot \nabla \psi_{q;\sigma }){â}_{p;\sigma }^{†}{â}_{q;\sigma } \end{array}$$ 21

Here, all operators except ∇ρ̂ yield scalars, while ∇ρ̂ is a vector.

### 2.4 Response Equations for Orbital-Rotation Parameters

In the presence of the perturbation, we write order corrections to the orbital-rotation parameters as

$$\kappa_{n}(t)=\sum_{\omega_{k}}\sum_{\alpha }\frac{d\kappa_{n}^{\omega_{k}}}{dF_{\alpha }^{\omega_{k}}}{|}_{F=0}F_{\alpha }^{\omega_{k}}e^{-i\omega_{k}t}+\frac{1}{2!}\sum_{\omega_{k},\omega_{l}}\sum_{\alpha ,\beta }\frac{d^{2}\kappa_{n}^{\omega_{k},\omega_{l}}}{dF_{\alpha }^{\omega_{k}}dF_{\beta }^{\omega_{l}}}{|}_{F=0}F_{\alpha }^{\omega_{k}}F_{\beta }^{\omega_{l}}e^{-i(\omega_{k}+\omega_{l})t}+\frac{1}{3!}\sum_{\omega_{k},\omega_{l},\omega_{m}}\sum_{\alpha ,\beta ,\gamma }\frac{d^{3}\kappa_{n}^{\omega_{k},\omega_{l},\omega_{m}}}{dF_{\alpha }^{\omega_{k}}dF_{\beta }^{\omega_{l}}dF_{\gamma }^{\omega_{m}}}{|}_{F=0}F_{\alpha }^{\omega_{k}}F_{\beta }^{\omega_{l}}F_{\gamma }^{\omega_{m}}e^{-i(\omega_{k}+\omega_{l}+\omega_{m})t}+...$$ 22

The first- and second-order corrections to the **κ**-vectors can then be constructed in terms of the Fourier amplitudes of the field as

$${\frac{d\kappa }{dF_{\beta }^{\omega_{k}}}|}_{F=0}=\left(\begin{array}{l} \frac{d\kappa_{n}}{dF_{\beta }^{\omega_{k}}}{|}_{F=0}\\ \frac{d\kappa_{n}^{*}}{dF_{\beta }^{-\omega_{k}}}{|}_{F=0} \end{array}\right)$$ 23

$${\frac{d^{2}\kappa }{dF_{\beta }^{\omega_{k}}dF_{\gamma }^{\omega_{l}}}|}_{F=0}=\left(\begin{array}{l} \frac{d^{2}\kappa_{n}}{dF_{\beta }^{\omega_{k}}dF_{\gamma }^{\omega_{l}}}{|}_{F=0}\\ \frac{d^{2}\kappa_{n}^{*}}{dF_{\beta }^{-\omega_{k}}dF_{\gamma }^{-\omega_{l}}}{|}_{F=0} \end{array}\right)$$ 24

In the working equations to come, we will most often use the matrix representation of the order corrections to the wave function parameters **κ**_α_^ω^, which is given as

$$\kappa_{\alpha }^{\omega }=\left(\begin{array}{ll} 0 & \frac{d\kappa^{\omega }}{dF_{\alpha }^{\omega }}\\ -(\frac{d\kappa^{-\omega }}{dF_{\alpha }^{-\omega }}{)}^{*} & 0 \end{array}\right)$$ 25

The determination of the order corrections to the quasi-energy requires the order corrections to the orbital rotation parameters, which are themselves solutions of the response equations. With the use of the time-dependent variational principle eq 9, we get the linear and quadratic response equations

$$\frac{d}{dF_{\beta }^{\omega_{k}}}\left(\frac{\partial Q_{T}}{\partial \kappa^{-\omega_{\sigma }}}\right){|}_{F=0}=\frac{\partial^{2}Q_{T}}{\partial \kappa^{-\omega_{\sigma }}\partial F_{\beta }^{\omega_{k}}}+\frac{\partial^{2}Q_{T}}{\partial \kappa^{-\omega_{\sigma }}\partial \kappa^{\omega_{k}}}\frac{d\kappa^{\omega_{k}}}{dF_{\beta }^{\omega_{k}}}=0$$ 26

$$\frac{d^{2}}{dF_{\gamma }^{\omega_{l}}dF_{\beta }^{\omega_{k}}}\left(\frac{\partial Q_{T}}{\partial \kappa^{-\omega_{\sigma }}}\right){|}_{F=0}=\frac{\partial^{3}Q_{T}}{\partial \kappa^{-\omega_{\sigma }}\partial \kappa^{\omega_{l}}\partial F_{\beta }^{\omega_{k}}}\frac{d\kappa^{\omega_{l}}}{dF_{\gamma }^{\omega_{l}}}+\frac{\partial^{3}Q_{T}}{\partial \kappa^{-\omega_{\sigma }}\partial \kappa^{\omega_{k}}\partial F_{\gamma }^{\omega_{l}}}\frac{d\kappa^{\omega_{k}}}{dF_{\beta }^{\omega_{k}}}+\frac{\partial^{3}Q_{T}}{\partial \kappa^{-\omega_{\sigma }}\partial \kappa^{\omega_{k}}\partial \kappa^{\omega_{l}}}\frac{d\kappa^{\omega_{k}}}{dF_{\beta }^{\omega_{k}}}\frac{d\kappa^{\omega_{l}}}{dF_{\gamma }^{\omega_{l}}}+\frac{\partial^{2}Q_{T}}{\partial \kappa^{-\omega_{\sigma }}\partial \kappa^{\omega_{k},\omega_{l}}}\frac{d^{2}\kappa^{\omega_{k},\omega_{l}}}{dF_{\beta }^{\omega_{k}}dF_{\gamma }^{\omega_{l}}}=0$$ 27

Solving for the second-order response vector in eqs 26 and 27 where we have symmetrized the response vectors in frequency variables ω_1_ and ω_2_, we get

$$\frac{d\kappa^{\omega_{k}}}{dF_{\beta }^{\omega_{k}}}=-{\left(\frac{\partial^{2}Q_{T}}{\partial \kappa^{-\omega_{\sigma }}\partial \kappa^{\omega_{k}}}\right)}^{-1}\frac{\partial^{2}Q_{T}}{\partial \kappa^{-\omega_{\sigma }}\partial F_{\beta }^{\omega_{k}}}$$ 28

$$\frac{d^{2}\kappa^{\omega_{k},\omega_{l}}}{dF_{\beta }^{\omega_{k}}dF_{\gamma }^{\omega_{l}}}={\left(\frac{\partial^{2}Q_{T}}{\partial \kappa^{-\omega_{\sigma }}\partial \kappa^{\omega_{k},\omega_{l}}}\right)}^{-1}\left[\frac{\partial^{3}Q_{T}}{\partial \kappa^{-\omega_{\sigma }}\partial \kappa^{\omega_{l}}\partial F_{\beta }^{\omega_{k}}}\frac{d\kappa^{\omega_{l}}}{dF_{\gamma }^{\omega_{l}}}+\frac{\partial^{3}Q_{T}}{\partial \kappa^{-\omega_{\sigma }}\partial \kappa^{\omega_{k}}\partial F_{\gamma }^{\omega_{l}}}\frac{d\kappa^{\omega_{k}}}{dF_{\beta }^{\omega_{k}}}+\frac{\partial^{3}Q_{T}}{\partial \kappa^{-\omega_{\sigma }}\partial \kappa^{\omega_{k}}\partial \kappa^{\omega_{l}}}\left(\frac{d\kappa^{\omega_{k}}}{dF_{\beta }^{\omega_{k}}}\frac{d\kappa^{\omega_{l}}}{dF_{\gamma }^{\omega_{l}}}+\frac{d\kappa^{\omega_{l}}}{dF_{\gamma }^{\omega_{l}}}\frac{d\kappa^{\omega_{k}}}{dF_{\beta }^{\omega_{k}}}\right)\right]$$ 29

The cubic response function, being part of the fourth-order correction to the time-averaged quasi-energy, can, by the 2*n* + 1 rule, be written in terms of the first- and second-order response of the wave-function parameters. A single element of the second hyperpolarizability is given by the expression

$$\gamma_{\alpha \beta \gamma \delta }(-\omega_{\sigma };\omega_{k},\omega_{l},\omega_{m})=\frac{d\kappa^{-\omega_{\sigma }}}{dF_{\alpha }^{-\omega_{\sigma }}}\frac{\partial^{4}Q_{T}}{\partial F\partial \kappa \partial \kappa \partial \kappa }\left[\frac{d\kappa^{\omega_{l}}}{dF_{\gamma }^{\omega_{l}}}\frac{d\kappa^{\omega_{m}}}{dF_{\delta }^{\omega_{m}}}+\frac{d\kappa^{\omega_{k}}}{dF_{\beta }^{\omega_{k}}}\frac{d\kappa^{\omega_{m}}}{dF_{\delta }^{\omega_{m}}}+\frac{d\kappa^{\omega_{k}}}{dF_{\beta }^{\omega_{k}}}\frac{d\kappa^{\omega_{l}}}{dF_{\gamma }^{\omega_{l}}}\right]+\frac{d\kappa^{-\omega_{\sigma }}}{dF_{\alpha }^{-\omega_{\sigma }}}\frac{\partial^{3}Q_{T}}{\partial F\partial \kappa \partial \kappa }\left[\frac{d^{2}\kappa^{\omega_{l},\omega_{m}}}{dF_{\gamma }^{\omega_{l}}dF_{\delta }^{\omega_{m}}}+\frac{d^{2}\kappa^{\omega_{k},\omega_{m}}}{dF_{\beta }^{\omega_{k}}dF_{\delta }^{\omega_{m}}}+\frac{d^{2}\kappa^{\omega_{k},\omega_{l}}}{dF_{\beta }^{\omega_{k}}dF_{\gamma }^{\omega_{l}}}\right]+\frac{d\kappa^{-\omega_{\sigma }}}{dF_{\alpha }^{-\omega_{\sigma }}}\frac{\partial^{3}Q_{T}}{\partial \kappa \partial \kappa \partial \kappa }\left[\frac{d\kappa^{\omega_{k}}}{dF_{\beta }^{\omega_{k}}}\frac{d^{2}\kappa^{\omega_{l},\omega_{m}}}{dF_{\gamma }^{\omega_{l}}dF_{\delta }^{\omega_{m}}}+\frac{d\kappa^{\omega_{l}}}{dF_{\gamma }^{\omega_{l}}}\frac{d^{2}\kappa^{\omega_{k},\omega_{m}}}{dF_{\beta }^{\omega_{k}}dF_{\delta }^{\omega_{m}}}+\frac{d\kappa^{\omega_{m}}}{dF_{\delta }^{\omega_{m}}}\frac{d^{2}\kappa^{\omega_{k},\omega_{l}}}{dF_{\beta }^{\omega_{k}}dF_{\gamma }^{\omega_{l}}}\right]+\frac{d\kappa^{-\omega_{\sigma }}}{dF_{\alpha }^{-\omega_{\sigma }}}\frac{\partial^{4}Q_{T}}{\partial \kappa \partial \kappa \partial \kappa \partial \kappa }\frac{d\kappa^{\omega_{k}}}{dF_{\beta }^{\omega_{k}}}\frac{d\kappa^{\omega_{l}}}{dF_{\gamma }^{\omega_{l}}}\frac{d\kappa^{\omega_{m}}}{dF_{\delta }^{\omega_{m}}}+\frac{\partial^{3}Q_{T}}{\partial F_{\alpha }^{\omega_{\sigma }}\partial \kappa \partial \kappa }\left[\frac{d\kappa^{\omega_{k}}}{dF_{\beta }^{\omega_{k}}}\frac{d^{2}\kappa^{\omega_{l},\omega_{m}}}{dF_{\gamma }^{\omega_{l}}dF_{\delta }^{\omega_{m}}}+\frac{d\kappa^{\omega_{l}}}{dF_{\gamma }^{\omega_{l}}}\frac{d^{2}\kappa^{\omega_{k},\omega_{m}}}{dF_{\beta }^{\omega_{k}}dF_{\delta }^{\omega_{m}}}+\frac{d\kappa^{\omega_{m}}}{dF_{\delta }^{\omega_{m}}}\frac{d^{2}\kappa^{\omega_{k},\omega_{l}}}{dF_{\beta }^{\omega_{k}}dF_{\gamma }^{\omega_{l}}}\right]+\frac{\partial^{4}Q_{T}}{\partial F\partial \kappa \partial \kappa \partial \kappa }\frac{d\kappa^{\omega_{k}}}{dF_{\beta }^{\omega_{k}}}\frac{d\kappa^{\omega_{l}}}{dF_{\gamma }^{\omega_{l}}}\frac{d\kappa^{\omega_{m}}}{dF_{\delta }^{\omega_{m}}}$$ 30

Importantly, the terms $\frac{\partial^{3}Q_{T}}{\partial \kappa \partial \kappa \partial \kappa }$ and $\frac{\partial^{4}Q_{T}}{\partial \kappa \partial \kappa \partial \kappa \partial \kappa }$ contain the third-order derivative of the energy (known as the cubic Hessian) and the fourth-order derivative of the energy (referred to as the quartic Hessian), respectively. To evaluate an element of the second-order nonlinear hyperpolarizability, one must contract these two tensors. The subsequent section delves into this process.

### 2.5 Tensor-Component Method for Isotropic Cubic Response

The cubic and quartic derivatives of the time-averaged quasi-energy with respect to the orbital-rotation parameters enter the expression for the cubic response function through contractions with first- and second-order orbital response vectors. These contractions transform the high-rank energy-derivative tensors into vectors of transformed Fock matrices^67,77–79^ . Consequently, their evaluation requires repeated construction of Fock matrices, two-electron integral contractions, and exchange–correlation kernel integrations. This step, therefore, constitutes the dominant computational bottleneck of cubic response calculations.

#### 2.5.1 The Tensor Component Contraction of the Cubic and Quartic Hessians

The contractions of the quartic and cubic electronic Hessians with orbital response vectors required in order to construct a single tensor element γ_αβγδ_(−ω_σ_; ω_*k*_, ω_*l*_, ω_*m*_) can be expressed as the vectors

$$\frac{\partial^{4}Q_{T}}{\partial \kappa^{4}}\frac{d\kappa }{dF_{\beta }^{\omega_{k}}}\frac{d\kappa }{dF_{\gamma }^{\omega_{l}}}\frac{d\kappa }{dF_{\delta }^{\omega_{m}}}=\left(\begin{array}{l} \sigma_{ai}^{\left(3\right)}+\lambda_{ai}^{\left(3\right)}+\tau_{ai}^{\left(3\right)}+f_{ai}^{\left(\omega_{k},\omega_{l},\omega_{m}\right)}\\ -(\sigma_{ia}^{\left(3\right)}+\lambda_{ia}^{\left(3\right)}+\tau_{ia}^{\left(3\right)}+f_{ia}^{\left(\omega_{k},\omega_{l},\omega_{m}\right)}) \end{array}\right)$$ 31

$$\frac{\partial^{3}Q_{T}}{\partial \kappa^{3}}\frac{d\kappa }{dF_{\beta }^{\omega_{k}}}\frac{d\kappa }{dF_{\gamma }^{\omega_{l}}}=\left(\begin{array}{l} \sigma_{ai}^{\left(2\right)}+\lambda_{ai}^{\left(2\right)}+f_{ai}^{\left(\omega_{k},\omega_{l}\right)}\\ -(\sigma_{ia}^{\left(2\right)}+\lambda_{ia}^{\left(2\right)}+f_{ia}^{\left(\omega_{k},\omega_{l}\right)}) \end{array}\right)$$ 32

The individual terms λ^(3)^, σ^(3)^, τ^(3)^, λ^(2)^, and σ^(2)^ entering the quartic and cubic contractions collect contributions arising from distinct permutations of the orbital-rotation operators and the associated auxiliary Fock matrices. Their explicit forms are

$$\begin{array}{cc} \lambda_{ai}^{\left(3\right)} & =\left[\kappa_{\beta }^{\omega_{k}},[\kappa_{\gamma }^{\omega_{l}},[\kappa_{\delta }^{\omega_{m}},f\right]+3f_{\delta }^{\omega_{m}}]+\left[\kappa_{\delta }^{\omega_{m}},[\kappa_{\gamma }^{\omega_{l}},f\right]+3f_{\gamma }^{\omega_{l}}]+3f^{\left(\omega_{l},\omega_{m}\right)}{]}_{ai}\\ \sigma_{ai}^{\left(3\right)} & =[\kappa_{\gamma }^{\omega_{l}},\left[\kappa_{\beta }^{\omega_{k}},[\kappa_{\delta }^{\omega_{m}},f\right]+3f_{\delta }^{\omega_{m}}]+\left[\kappa_{\delta }^{\omega_{m}},[\kappa_{\beta }^{\omega_{k}},f\right]+3f_{\beta }^{\omega_{k}}]+3f^{\left(\omega_{k},\omega_{m}\right)}{]}_{ai}\\ \tau_{ai}^{\left(3\right)} & =\left[\kappa_{\delta }^{\omega_{m}},[\kappa_{\gamma }^{\omega_{l}},[\kappa_{\beta }^{\omega_{k}},f\right]+3f_{\beta }^{\omega_{k}}]+\left[\kappa_{\beta }^{\omega_{k}},[\kappa_{\gamma }^{\omega_{l}},f\right]+3f_{\gamma }^{\omega_{l}}]+3f^{\left(\omega_{k},\omega_{l}\right)}{]}_{ai} \end{array}$$ 33

$$\begin{array}{c} \sigma_{ai}^{\left(2\right)}=[\kappa_{\beta }^{\omega_{k}},[\kappa_{\gamma }^{\omega_{l}},f]+2f_{\gamma }^{\omega_{l}}{]}_{ai}\\ \lambda_{ai}^{\left(2\right)}=[\kappa_{\gamma }^{\omega_{l}},[\kappa_{\beta }^{\omega_{k}},f]+2f_{\beta }^{\omega_{k}}{]}_{ai} \end{array}$$ 34

The presence of parentheses in these expressions implies permutation with respect to operator–frequency pairs, ensuring full symmetry of the response functions. At the level of Kohn–Sham density functional theory, the transformed Fock matrices entering these contractions are built from auxiliary Fock matrices of increasing perturbation order. Up to the third order, these auxiliary Fock matrices are given by the following tensor contractions:^51^

$$f_{ai;\alpha ;\sigma }^{\omega }=\sum_{p,q}g_{aipq}D_{pq;\alpha ;\sigma }^{\omega }+\sum_{\sigma^{\prime }}\sum_{p,q}v_{xc,pq,ai;\sigma ,\sigma \prime }^{\left(1\right)}D_{pq;\alpha ;\sigma \prime }^{\omega }$$ 35

$$f_{ai;\beta ,\gamma ;\sigma }^{\omega_{k},\omega_{l}}=\sum_{p,q}g_{aipq}D_{pq;\beta ,\gamma ;\sigma }^{\omega_{k},\omega_{l}}+\sum_{\sigma^{\prime }}\sum_{p,q}v_{xc,pq,ai;\sigma ,\sigma \prime }^{\left(1\right)}D_{pq;\beta ,\gamma ;\sigma \prime }^{\omega_{k},\omega_{l}}+\sum_{\sigma^{\prime },\sigma^{\prime \prime }}\sum_{p,q,t,u}v_{xc,pq,tu,ai;\sigma ,\sigma \prime ,\sigma \prime \prime }^{\left(2\right)}D_{pq;\beta ;\sigma \prime }^{\omega_{k}}D_{tu;\gamma ;\sigma \prime \prime }^{\omega_{l}}$$ 36

$$f_{ai;\beta ,\gamma ,\delta ;\sigma }^{\omega_{k},\omega_{l},\omega_{m}}=\underset{p,q}{\sum }g_{aipq}D_{pq;\beta ,\gamma ,\delta ;\sigma }^{\omega_{k},\omega_{l},\omega_{m}}+\underset{\sigma^{\prime }}{\sum }\underset{p,q}{\sum }v_{xc,pq,ai;\sigma ,\sigma \prime }^{\left(1\right)}D_{pq;\beta ,\gamma ,\delta ;\sigma \prime }^{\omega_{k},\omega_{l},\omega_{m}}+\underset{\sigma^{\prime },\sigma^{\prime \prime }}{\sum }\underset{p,q,t,u}{\sum }v_{xc,pq,tu,ai;\sigma ,\sigma \prime ,\sigma \prime \prime }^{\left(2\right)}\left(D_{pq;\beta ,\gamma ;\sigma \prime }^{\omega_{k},\omega_{l}}D_{tu;\delta ;\sigma \prime \prime }^{\omega_{m}}+D_{pq;\alpha ,\gamma ;\sigma \prime }^{\omega_{1},\omega_{3}}D_{tu;\beta ;\sigma \prime \prime }^{\omega_{2}}+D_{pq;\beta ,\gamma ;\sigma \prime }^{\omega_{2},\omega_{3}}D_{tu;\alpha ;\sigma \prime \prime }^{\omega_{1}}\right)+\underset{\sigma^{\prime },\sigma^{\prime \prime },\sigma^{\prime \prime \prime }}{\sum }\underset{p,q,t,u,r,s}{\sum }v_{xc,pq,tu,rs,ai;\sigma ,\sigma \prime ,\sigma \prime \prime ,\sigma \prime \prime \prime }^{\left(3\right)}D_{pq;\beta ;\sigma \prime }^{\omega_{k}}D_{tu;\gamma ;\sigma \prime \prime }^{\omega_{l}}D_{rs;\delta ;\sigma \prime \prime \prime }^{\omega_{m}}$$ 37

with the perturbed density matrices defined through nested commutators as

$$\begin{array}{c} D_{pq;\alpha ;\sigma }^{\omega_{k}}=\langle 0|[{\mathrm{\kappa ̂}}_{\alpha }^{\omega_{k}},{Ê}_{pq;\sigma }]|0\rangle \\ D_{pq;\alpha \beta ;\sigma }^{\omega_{k},\omega_{l}}=\langle 0|[{\mathrm{\kappa ̂}}_{\alpha }^{\omega_{k}},[{\mathrm{\kappa ̂}}_{\beta }^{\omega_{l}},{Ê}_{pq;\sigma }]]|0\rangle \\ D_{pq;\alpha \beta \gamma ;\sigma }^{\omega_{k},\omega_{l},\omega_{m}}=\langle 0|[{\mathrm{\kappa ̂}}_{\alpha }^{\omega_{k}},[{\mathrm{\kappa ̂}}_{\beta }^{\omega_{l}},[{\mathrm{\kappa ̂}}_{\gamma }^{\omega_{m}},{Ê}_{pq;\sigma }]]]|0\rangle \end{array}$$ 38

Here, *g* is the two-electron integral tensor and *v*_xc_^(2)^ and *v*_xc_^(3)^ denote the second- and third-order exchange–correlation kernels, respectively. For an approximate exchange–correlation functional from eq 19, the second- and third-order exchange–correlation kernel contractions are explicitly given by the expressions

∑σ′,σ″∑p,q,t,uvxc,pq,tu,ai;σ,σ′,σ″(2)Dpq;α;σ′ω1Dtu;β;σ″ω2=∑σ′,σ″⟨0|[âiσ†âaσ,∫(∂3exc∂Xσ∂Yσ′∂Zσ″X̂σ+∂3exc∂∇ρσ∂Yσ′∂Zσ″∇ρ̂σ)ΓYZα,β;σ′,σ″d3r]|0⟩+∑σ′,σ″⟨0|[âiσ†âaσ,∫(∂3exc∂Xσ∂∇νρσ′∂Zσ″X̂σ+∂3exc∂∇μρσ∂∇νρσ′∂Zσ″∇μρ̂σ)ΓZν;α,β;σ′,σ″d3r]|0⟩+∑σ′,σ″⟨0|[âiσ†âaσ,∫(∂3exc∂Xσ∂∇μρσ′∂∇νρσ″X̂σ+∂3exc∂∇ξρσ∂∇μρσ′∂∇nuρσ″∇ξρ̂σ)Γμν;α,β;σ′,σ″d3r]|0⟩ 39

∑σ′,σ″,σ″′∑p,q,t,u,r,svxc,pq,tu,rs,ai;σ,σ′,σ″,σ″′(3)Dpq;α;σ′ω1Dtu;β;σ″ω2Drs;γ;σ″′ω3=∑α,β,γδx,y,z∑σ′,σ″σ″′⟨0|[âiσ†âaσ,∫(∂4exc∂Wσ∂Xσ′∂Yσ″∂Zσ″′Ŵσ+∂4exc∂∇δρσ∂Xσ′∂Yσ″∂Zσ″′∇δρ̂σ)ΠXYZσ′,σ″,σ″′(r)d3r]|0⟩+⟨0|[âiσ†âaσ,∫(∂4exc∂Wσ∂∇αρσ′∂Xσ″∂Yσ″′Ŵσ+∂4exc∂∇δρσ∂∇αρσ′∂Xσ″∂Yσ″′∇δρ̂σ)ΠXYμ;σ′,σ″,σ″′(r)d3r]|0⟩+⟨0|[âiσ†âaσ,∫(∂4exc∂Wσ∂∇αρσ′∂∇βρσ″∂Xσ″′Ŵσ+∂4exc∂∇δρσ∂∇αρσ′∂∇βρσ″∂Xσ″′∇δρ̂σ)ΠXμν;σ′,σ″,σ″′(r)d3r]|0⟩+⟨0|[âiσ†âaσ,∫(∂4exc∂Wσ∂∇αρσ′∂∇βρσ″∂∇γρσ″′Ŵσ+∂4exc∂∇δρσ∂∇αρσ′∂∇βρσ″∂∇γρσ″′∇δρ̂σ)Πμνξ;σ′,σ″,σ″′(r)d3r]|0⟩ 40

Here, *X̂*, *Ŷ*, *Ẑ*, and *Ŵ*, which belong to the set of scalar operators {*ρ̂*, *τ̂*, and ∇^2^ *ρ̂*} are given in eq 21. In order to make use of the linearity of the exchange–correlation kernel in terms of the perturbed densities,^51^ the exchange-correlation kernel has been written in terms of products of perturbed densities, denoted as Γ. The exchange–correlation density *e*_xc_ is a scalar, and when one takes its derivative with respect to the density gradient ∇ρ, which is a vector, the resulting term itself becomes a vector. Higher-order derivatives with respect to the density gradient will correspondingly result in tensors. The final contraction between the derivative tensors and the perturbed densities and density gradients will always be scalar. The first term in the first row contains no derivatives with respect to the density gradient and is multiplied with ΓYZα,β;σ′,σ″ω1,ω2 , given in eq 41, which is a scalar. The second term in the first row contains a derivative with respect to the density gradient and is hence a vector; this vector is then contracted with the gradient operator (also a vector) and the scalar density product ΓYZα,β;σ′,σ″ω1,ω2 . The second row contains one density gradient derivative; the resulting vector is to be contracted with ΓZμ;α,β;σ′,σ″ω1,ω2 , (a vector) given by in eq 42. In conclusion, the last row contains derivatives with two or three density gradient derivatives, which result in rank two and rank three tensors that are contracted with the tensor Γ_μν;α,β;σ′,σ*″*_^ω_1_,ω_2_^, given in eq 43. In these equations, μ, ν, and ξ are the spatial components of the gradients and α and β are the spatial components of the one, two, and three-photon transition moment vectors in the response equations of eqs 26 and 27. The explicit expressions for the Γ density products are given as

ΓYZα,β;σ′,σ″ω1,ω2=⟨0|[κ̂αω1,Ŷσ′]|0⟩⟨0|[κ̂βω2,Ẑσ″]|0⟩+⟨0|[κ̂αω1,Ẑσ′]|0⟩⟨0|[κ̂βω2,Ŷσ″]|0⟩ 41

ΓZμ;α,β;σ′,σ″ω1,ω2=⟨0|[κ̂αω1,Ẑσ′]|0⟩⟨0|[κ̂βω2,∇μρ̂σ″]|0⟩+⟨0|[κ̂αω1,∇μρ̂σ′]|0⟩⟨0|[κ̂βω2,Ẑσ″]|0⟩ 42

$$\Gamma_{\mu \nu ;\alpha ,\beta ;\sigma \prime ,\sigma \prime \prime }^{\omega_{1},\omega_{2}}=\langle 0|[{\mathrm{\kappa ̂}}_{\alpha }^{\omega_{1}},\nabla_{\mu }{\mathrm{\rho ̂}}_{\sigma \prime }]|0\rangle \langle 0|[{\mathrm{\kappa ̂}}_{\beta }^{\omega_{2}},\nabla_{\nu }{\mathrm{\rho ̂}}_{\sigma \prime \prime }]|0\rangle$$ 43

The three-times transformed Fock matrix of eq 37 requires the contraction of the cubic exchange–correlation kernel with the perturbed densities. The contraction can be written as products of perturbed densities Π, which are themselves written in terms of the generalized densities product found in the quadratic exchange–correlation kernel Γ of eqs 41–43. The logic is otherwise similar to the formulation of the second-order exchange–correlation kernel of eq 39. Here, ΠXYZσ′,σ″,σ″′ω1,ω2,ω3 is a scalar, ΠXYν;σ′,σ″,σ″′ω1,ω2,ω3 is a vector, ΠXμν;α,β,γ;σ′,σ″,σ″′ω1,ω2,ω3 is a rank-two tensor, and Π_νμξ;σ′,σ″,σ*‴*_^ω_1_,ω_2_,ω_3_^ is a rank-three tensor.

ΠXYZσ′,σ″,σ″′ω1,ω2,ω3=ΓXYα,β;σ′,σ″ω1,ω2⟨0|[κ̂γω3,Ẑσ″′]|0⟩+ΓXZα,β;σ′,σ″ω1,ω2⟨0|[κ̂γω3,Ŷσ″′]|0⟩+ΓYZα,β;σ′,σ″ω1,ω2⟨0|[κ̂γω3,X̂σ″′]|0⟩ 44

ΠXYν;σ′,σ″,σ″′ω1,ω2,ω3=ΓXYβ,γ;σ′,σ″′ω2,ω3⟨0|[κ̂αω1,∇μρ̂σ′]|0⟩+ΓXYα,γ;σ′,σ″′ω1,ω3⟨0|[κ̂βω2,∇μρ̂σ″]|0⟩+ΓXYα,β;σ′,σ″ω1,ω2⟨0|[κ̂γω3,∇μρ̂σ″′]|0⟩ 45

ΠXμν;α,β,γ;σ′,σ″,σ″′ω1,ω2,ω3=Γμν;β,γ;σ′,σ″ω2,ω3⟨0|[κ̂αω1,X̂σ″′]|0⟩+Γμν;α,γ;σ′,σ″ω1,ω3⟨0|[κ̂βω2,X̂σ″′]|0⟩+Γμν;α,β;σ′,σ″ω1,ω2⟨0|[κ̂γω3,X̂σ″′]|0⟩ 46

$$\Pi_{\nu \mu \xi ;\sigma \prime ,\sigma \prime \prime ,\sigma \prime \prime \prime }^{\omega_{1},\omega_{2},\omega_{3}}=\langle 0|[{\mathrm{\kappa ̂}}_{\alpha }^{\omega_{1}},\nabla_{\mu }{\mathrm{\rho ̂}}_{\sigma \prime }]|0\rangle \langle 0|[{\mathrm{\kappa ̂}}_{\beta }^{\omega_{2}},\nabla_{\nu }{\mathrm{\rho ̂}}_{\sigma \prime \prime }]|0\rangle \langle 0|[{\mathrm{\kappa ̂}}_{\gamma }^{\omega_{3}},\nabla_{\xi }{\mathrm{\rho ̂}}_{\sigma \prime }]|0\rangle$$ 47

### 2.6 Tensor-Averaging Method for Isotropic Cubic Response

In Section 2.1, we showed that for third-harmonic generation in an isotropic medium, the macroscopic response is governed by a single scalar constructed from the molecular second hyperpolarizability, obtained by orientational averaging of the Cartesian tensor γ_αβγδ_(−ω_σ_;ω_1_, ω_2_, and ω_3_). More generally, the tensor-averaging strategy employed in the present work applies whenever the target orientationally averaged observable can be written as a linear contraction of the underlying Cartesian tensor components,

$$\mathrm{\gamma ̅}(-\omega_{\sigma };\omega_{1},\omega_{2},\omega_{3})=\sum_{\alpha \beta \gamma \delta }C_{\alpha \beta \gamma \delta }\gamma_{\alpha \beta \gamma \delta }(-\omega_{\sigma };\omega_{1},\omega_{2},\omega_{3})$$ 48

where the coefficient *C*_αβγδ_ defines the particular orientational average or effective scalar quantity of interest. In the present work, the specific choice of *C*_αβγδ_ corresponds to the orientationally averaged observable relevant to coherent third-harmonic generation in an isotropic medium. For this observable, the tensor-averaged and conventional tensor-component routes are formally equivalent, since the isotropically averaged scalar is obtained by an analytic linear contraction of the same underlying cubic-response expressions. The tensor-averaged formulation employed here, therefore, does not introduce any additional approximation for the target quantity; what is not retained is only the set of individual Cartesian tensor elements themselves, since these are not needed for the observable of interest. At the same time, this also clarifies that the present scalar quantity should not be interpreted as a universal descriptor for all third-order nonlinear optical observables, in particular, not for observables involving quadratic combinations of tensor elements, such as those relevant to third-harmonic scattering (THS).

In a straightforward implementation, one would first compute all required Cartesian components of the cubic response function explicitly and only afterward form the required isotropic combination. However, the dominant computational cost in such a calculation arises from the repeated contractions of the cubic and quartic electronic Hessians with response vectors, cf. eqs 31–34 since these require repeated calculations of two-electron integrals and exchange–correlation kernel integrations.

These Hessian contractions can be written in terms of transformed Fock matrices that are linear transformations of the perturbed densities. Symbolically, we may denote this as

f=F[D]

where F collects the Hartree and exchange–correlation contributions defined in eqs 35–([Disp-formula eq37]). Crucially, F is *linear* in the density,

$$F\left[\sum_{t}D_{t}\right]=\sum_{t}F[D_{t}]$$ 49

so that the same Fock-building machinery is used for each tensor element, only with different density perturbations as input.

The tensor-averaging strategy exploited in this work is to apply the isotropic contraction before the Fock construction, at the level of the perturbed densities and generalized density products (Γ and Π tensors). That is, instead of forming isotropic combinations of many Fock matrices ***f*** [***D***_*t*_] a posteriori, we first build compounded (tensor-averaged) densities, such as Γ and Π in eqs 53–55 and 63–66, and then apply the linear map F only once. In the following subsections, we make this procedure explicit by deriving the isotropically compounded exchange–correlation kernel contractions entering the quartic- and cubic-Hessian terms. This yields tensor-averaged transformed Fock matrices that directly generate the isotropic cubic response, while requiring the minimum number of Fock builds. To solve the response equations in eqs 26 and 27, we employ an iterative subspace method with symmetrized trial vectors, following the subspace procedures introduced by Kauczor et al.^53,54^ In this approach, the solution of the response equations proceeds through repeated applications of the electronic Hessian to a set of trial vectors spanning a reduced response space. Each Hessian-vector contraction is realized through the construction of an auxiliary transformed Fock matrix, which constitutes the dominant computational cost of the response solver. During the iterative procedure, these auxiliary Fock matrices are generated solely to expand and refine the response subspace. Once the response vectors have converged, however, a subset of the transformed Fock matrices required for higher-order Hessian contractions can be constructed as linear combinations of the auxiliary Fock matrices associated with the subspace vectors. We refer to this reuse of subspace-generated auxiliary Fock matrices for the evaluation of higher-order response contributions as subspace extraction. This strategy eliminates the need for previously calculated redundant auxiliary Fock matrices. In the following, the term tensor-averaging method is used to denote the combined application of the linearity of the Fock construction and the subspace extraction method.

#### 2.6.1 Tensor-Averaged Contraction of the Cubic and Quartic Hessians

Having established that the orientationally averaged second hyperpolarizability can be obtained by applying the rotational average directly to the perturbed densities, we now turn to the corresponding contractions of the cubic and quartic electronic Hessians. In the conventional Cartesian formulation, these Hessian contractions are evaluated separately for every tensor element of γ_αβγδ_, even though only their particular isotropic combination contributes to the THG response in an isotropic medium. This leads to a proliferation of redundant transformed Fock matrices. Because the Fock-building operator F is linear in the perturbed density, eq 49, the quartic- and cubic-Hessian terms can be expressed in terms of a small number of compounded density quantities. Applying F to these isotropically contracted densities yields directly the transformed Fock matrices required for the isotropic cubic response, eliminating the need to evaluate each Cartesian component individually. In practice, this means that instead of constructing the quartic-Hessian contraction in eq 31 for all Cartesian index permutations and inserting them into eq 5, we derive a single, tensor-averaged contraction expressed entirely in terms of compounded Fock matrices. These isotropically averaged transformed Fock matrices can be written as

$$\left\langle \frac{d\kappa^{-\omega_{\sigma }}}{dF_{\alpha }^{-\omega_{\sigma }}}\cdot \frac{\partial^{4}Q_{T}}{\partial \kappa^{4}}\frac{d\kappa }{dF_{\beta }^{\omega_{k}}}\frac{d\kappa }{dF_{\gamma }^{\omega_{l}}}\frac{d\kappa }{dF_{\delta }^{\omega_{m}}}\right\rangle =\sum_{\alpha ,\beta }^{x,y,z}\left(\frac{d\kappa^{-\omega_{\sigma }}}{dF_{\alpha }^{-\omega_{\sigma }}}\cdot \left[\begin{array}{l} ([\kappa_{\beta }^{\omega },\Theta_{\alpha \beta }+3f_{\alpha \beta }^{N}]+f_{\alpha }^{M}{)}_{ia}\\ -([\kappa_{\beta }^{\omega },\Theta_{\alpha \beta }+3f_{\alpha \beta }^{N}]+f_{\alpha }^{M}{)}_{ai} \end{array}\right]\right)$$ 50

$$\left\langle \frac{d\kappa^{-\omega_{\sigma }}}{dF_{\alpha }^{-\omega_{\sigma }}}\cdot \frac{\partial^{3}Q_{T}}{\partial \kappa^{3}}\frac{d\kappa }{dF_{\beta }^{\omega_{k}}}\frac{d\kappa }{dF_{\gamma }^{\omega_{l}}}\right\rangle =\sum_{\alpha }^{x,y,z}\left(\frac{d\kappa^{-\omega_{\sigma }}}{dF_{\alpha }^{-\omega_{\sigma }}}\cdot \left[\begin{array}{l} (\zeta_{\alpha }+f_{\alpha }^{L}{)}_{ai}\\ -(\zeta_{\alpha }+f_{\alpha }^{L}{)}_{ia} \end{array}\right]\right)$$ 51

for the quartic and cubic contractions of the electronic Hessian. Here, the indices α and β denote the Cartesian components of the property gradients entering the response equations, while **Θ**_αβ_ and **ζ**_α_ collect the contributions that do not involve explicit exchange–correlation kernels or two-electron integral contractions in eqs 33 and 34 for auxiliary Fock matrices exceeding second order. The quantities ***f***_αβ_^*N*^, ***f***_α_^*L*^, and ***f***_α_^*M*^ are compounded Fock matrices: they are obtained by first forming the appropriate isotropic combinations of the underlying perturbed densities (and generalized density products) and only then applying the linear Fock map F of eq 49. Thus, the orientational averaging needed to extract the THG-relevant quantity is carried out once at the level of the perturbed densities and their Fock images, and the resulting effective Fock matrices in eq 50 directly generate the isotropic cubic response with the minimum number of Fock builds. The second-order exchange–correlation contribution to the compounded Fock matrices ***f***_αβ_^*N*^, ***f***_α_^*L*^ are with the use of eq 39 is given by

vxc,pq,tu,ai;σ,σ′,σ″N=∑σ′,σ″⟨0|[âiσ†âaσ,∫(∂3exc∂Xσ∂Yσ′∂Zσ″X̂σ+∂3exc∂∇ρσ∂Yσ′∂Zσ″∇ρ̂σ)ΓYZα,β;σ′,σ″Nd3r]|0⟩+∑σ′,σ″⟨0|[âiσ†âaσ,∫(∂3exc∂Xσ∂∇νρσ′∂Zσ″X̂σ+∂3exc∂∇μρσ∂∇νρσ′∂Zσ″∇μρ̂σ)ΓZν;α,β;σ′,σ″Nd3r]|0⟩+∑σ′,σ″⟨0|[âiσ†âaσ,∫(∂3exc∂Xσ∂∇μρσ′∂∇νρσ″X̂σ+∂3exc∂∇ξρσ∂∇μρσ′∂∇nuρσ″∇ξρ̂σ)Γμν;α,β;σ′,σ″Nd3r]|0⟩ 52

where the compounded densities are defined with the use of eqs 41 and 43 as

ΓYZαβ;σ″,σ″′N=6ΓYZαβ;σ″,σ″′(ω,ω)+6ΓYZβα;σ″,σ″′(ω,ω)+6δαβ∑ϵΓYZϵϵ;σ″,σ″′(ω,ω) 53

ΓZμ;αβ;σ″,σ″′N=6ΓZμ;αβ;σ″,σ″′(ω,ω)+6ΓZμ;βα;σ″,σ″′(ω,ω)+6δαβ∑ϵΓZμ;ϵϵ;σ″,σ″′(ω,ω) 54

$$\Gamma_{\mu \nu ;\alpha \beta ;\sigma \prime \prime ,\sigma \prime \prime \prime }^{N}=6\Gamma_{\mu \nu ;\alpha \beta ;\sigma \prime \prime ,\sigma \prime \prime \prime }^{\left(\omega ,\omega \right)}+6\Gamma_{\mu \nu ;\beta \alpha ;\sigma \prime \prime ,\sigma \prime \prime \prime }^{\left(\omega ,\omega \right)}+6\delta_{\alpha \beta }\underset{\epsilon }{\sum }\Gamma_{\mu \nu ;\epsilon \epsilon ;\sigma \prime \prime ,\sigma \prime \prime \prime }^{\left(\omega ,\omega \right)}$$ 55

Likewise, we get

vxc,pq,tu,ai;σ,σ′,σ″L=∑σ′,σ″⟨0|[âiσ†âaσ,∫(∂3exc∂Xσ∂Yσ′∂Zσ″X̂σ+∂3exc∂∇ρσ∂Yσ′∂Zσ″∇ρ̂σ)ΓYZα,β;σ′,σ″Ld3r]|0⟩+∑σ′,σ″⟨0|[âiσ†âaσ,∫(∂3exc∂Xσ∂∇νρσ′∂Zσ″X̂σ+∂3exc∂∇μρσ∂∇νρσ′∂Zσ″∇μρ̂σ)ΓZν;α,β;σ′,σ″Ld3r]|0⟩+∑σ′,σ″⟨0|[âiσ†âaσ,∫(∂3exc∂Xσ∂∇μρσ′∂∇νρσ″X̂σ+∂3exc∂∇ξρσ∂∇μρσ′∂∇nuρσ″∇ξρ̂σ)Γμν;α,β;σ′,σ″Ld3r]|0⟩ 56

where the compounded densities are given in terms of the second-order perturbed densities of eqs 60 and 61 as

ΓYZσ′,σ″L=∑β(⟨0|[κ̂βω,Ŷσ″]|0⟩Zαβ;σ′L+(⟨0|[κ̂βω,Ẑσ″]|0⟩Yαβ;σ′L) 57

ΓZμ;σ′,σ″L=∑β(⟨0|[κ̂βω,∇μρ̂σ″]|0⟩Zαβ;σ′L+(⟨0|[κ̂βω,Ẑσ″]|0⟩∇μραβ;σ′L) 58

$$\Gamma_{\mu \nu ;\sigma \prime ,\sigma \prime \prime }^{L}=\sum_{\beta }\langle 0|[{\mathrm{\kappa ̂}}_{\beta }^{\omega },\nabla_{\mu }{\mathrm{\rho ̂}}_{\sigma \prime \prime }]|0\rangle \nabla_{\nu }\rho_{\alpha \beta ;\sigma \prime }^{L}$$ 59

$$X_{\alpha \beta ;\sigma \prime }^{L}=6\langle 0|[{\mathrm{\kappa ̂}}_{\alpha }^{\omega },[{\mathrm{\kappa ̂}}_{\beta }^{\omega },{\mathrm{X̂}}_{\sigma \prime }]]|0\rangle +6\langle 0|[{\mathrm{\kappa ̂}}_{\beta }^{\omega },[{\mathrm{\kappa ̂}}_{\alpha }^{\omega },{\mathrm{X̂}}_{\sigma \prime }]]|0\rangle +6\delta_{\alpha \beta }\sum_{\epsilon }^{x,y,z}\langle 0|[{\mathrm{\kappa ̂}}_{\epsilon }^{\omega },[{\mathrm{\kappa ̂}}_{\epsilon }^{\omega },{\mathrm{X̂}}_{\sigma \prime }]]|0\rangle$$ 60

$$\nabla_{\mu }\rho_{\alpha \beta ;\sigma \prime }^{L}=6\langle 0|[[{\mathrm{\kappa ̂}}_{\beta }^{\omega },\nabla_{\mu }{\mathrm{\rho ̂}}_{\sigma \prime }]]|0\rangle +6\langle 0|[{\mathrm{\kappa ̂}}_{\beta }^{\omega },[{\mathrm{\kappa ̂}}_{\alpha }^{\omega },\nabla_{\mu }{\mathrm{\rho ̂}}_{\sigma \prime }]]|0\rangle +6\delta_{\alpha \beta }\sum_{\epsilon }^{x,y,z}\langle 0|[{\mathrm{\kappa ̂}}_{\epsilon }^{\omega },[{\mathrm{\kappa ̂}}_{\epsilon }^{\omega },\nabla_{\mu }{\mathrm{\rho ̂}}_{\sigma \prime }]]|0\rangle$$ 61

The compounded third-order exchange–correlation tensor contraction required to construct ***f*** ^*M*^ in eq 50 is then using eq 40 given by

$$v_{xc,pq,tu,rs,ai;\sigma ,\sigma \prime ,\sigma \prime \prime ,\sigma \prime \prime \prime }^{M}=\sum_{\alpha ,\beta ,\gamma \delta }^{x,y,z}\sum_{\sigma^{\prime },\sigma^{\prime \prime }\sigma^{\prime \prime \prime }}\langle 0|\left[{â}_{i\sigma }^{†}{â}_{a\sigma },\;\int \left(\frac{\partial^{4}e_{\mathrm{xc}}}{\partial W_{\sigma }\partial X_{\sigma \prime }\partial Y_{\sigma \prime \prime }\partial Z_{\sigma \prime \prime \prime }}{Ŵ}_{\sigma }+\frac{\partial^{4}e_{\mathrm{xc}}}{\partial \nabla_{\delta }\rho_{\sigma }\partial X_{\sigma \prime }\partial Y_{\sigma \prime \prime }\partial Z_{\sigma \prime \prime \prime }}\nabla_{\delta }{\mathrm{\rho ̂}}_{\sigma }\right)\Pi_{\sigma \prime ,\sigma \prime \prime ,\sigma \prime \prime \prime }^{M}(r)d^{3}r\right]|0\rangle +\langle 0|\left[{â}_{i\sigma }^{†}{â}_{a\sigma },\;\int \left(\frac{\partial^{4}e_{\mathrm{xc}}}{\partial W_{\sigma }\partial \nabla_{\alpha }\rho_{\sigma \prime }\partial X_{\sigma \prime \prime }\partial Y_{\sigma \prime \prime \prime }}{Ŵ}_{\sigma }+\frac{\partial^{4}e_{\mathrm{xc}}}{\partial \nabla_{\delta }\rho_{\sigma }\partial \nabla_{\alpha }\rho_{\sigma \prime }\partial X_{\sigma \prime \prime }\partial Y_{\sigma \prime \prime \prime }}\nabla_{\delta }{\mathrm{\rho ̂}}_{\sigma }\right)\Pi_{\mu ;\sigma \prime ,\sigma \prime \prime ,\sigma \prime \prime \prime }^{M}(r)d^{3}r\right]|0\rangle +\langle 0|\left[{â}_{i\sigma }^{†}{â}_{a\sigma },\;\int \left(\frac{\partial^{4}e_{\mathrm{xc}}}{\partial W_{\sigma }\partial \nabla_{\alpha }\rho_{\sigma \prime }\partial \nabla_{\beta }\rho_{\sigma \prime \prime }\partial X_{\sigma \prime \prime \prime }}{Ŵ}_{\sigma }+\frac{\partial^{4}e_{\mathrm{xc}}}{\partial \nabla_{\delta }\rho_{\sigma }\partial \nabla_{\alpha }\rho_{\sigma \prime }\partial \nabla_{\beta }\rho_{\sigma \prime \prime }\partial X_{\sigma \prime \prime \prime }}\nabla_{\delta }{\mathrm{\rho ̂}}_{\sigma }\right)\Pi_{\mu \nu ;\sigma \prime ,\sigma \prime \prime ,\sigma \prime \prime \prime }^{M}(r)d^{3}r\right]|0\rangle +\langle 0|\left[{â}_{i\sigma }^{†}{â}_{a\sigma },\;\int \left(\frac{\partial^{4}e_{\mathrm{xc}}}{\partial W_{\sigma }\partial \nabla_{\alpha }\rho_{\sigma \prime }\partial \nabla_{\beta }\rho_{\sigma \prime \prime }\partial \nabla_{\gamma }\rho_{\sigma \prime \prime \prime }}{Ŵ}_{\sigma }+\frac{\partial^{4}e_{\mathrm{xc}}}{\partial \nabla_{\delta }\rho_{\sigma }\partial \nabla_{\alpha }\rho_{\sigma \prime }\partial \nabla_{\beta }\rho_{\sigma \prime \prime }\partial \nabla_{\gamma }\rho_{\sigma \prime \prime \prime }}\nabla_{\delta }{\mathrm{\rho ̂}}_{\sigma }\right)\Pi_{\mu \nu \xi ;\sigma \prime ,\sigma \prime \prime ,\sigma \prime \prime \prime }^{M}(r)d^{3}r\right]|0\rangle$$ 62

where the compounded Π densities can be written in terms of the compounded Γ densities of eqs 53–55 as

ΠXYZα;σ′,σ″,σ″′M=∑β(⟨0|[κ̂βω,X̂σ″′]|0⟩ΓYZαβ;σ′,σ″N+⟨0|[κ̂βω,Ŷσ″′]|0⟩ΓXZαβ;σ′,σ″N+⟨0|[κ̂βω,Ẑσ″′]|0⟩ΓYXαβ;σ′,σ″N) 63

ΠXYμ;α;σ′,σ″,σ″′M=∑β⟨0|[κ̂βω,∇μρ̂σ″′]|0⟩ΓXYαβ;σ′,σ″N 64

ΠXμν;α;σ′,σ″,σ″′M=∑β⟨0|[κ̂βω,X̂σ″′]|0⟩Γμν;αβ;σ′,σ″N 65

$$\Pi_{\mu \nu \xi ;\alpha ;\sigma \prime ,\sigma \prime \prime ,\sigma \prime \prime \prime }^{M}=\sum_{\beta }\langle 0|[{\mathrm{\kappa ̂}}_{\beta }^{\omega },\nabla_{\xi }{\mathrm{\rho ̂}}_{\sigma \prime \prime \prime }]|0\rangle \Gamma_{\mu \nu ;\alpha \beta ;\sigma \prime ,\sigma \prime \prime }^{N}$$ 66

In the next section, we will illustrate an application and discuss the performance of the tensor-average method.

## 3 Results and Discussion

To demonstrate the applicability of the developed isotropic cubic-response formalism, the results are organized to highlight two key aspects. First, frequency-dispersed THG spectra are analyzed for the prototypical donor–acceptor systems OTBP and ODBP to establish the connection between electronic structure and resonance-enhanced nonlinear response. Second, the methodology is applied to molecular aggregates of OTBP to elucidate how specific packing motifs govern aggregation-induced enhancement and saturation behavior. All electronic-structure calculations and nonlinear optical response calculations were performed using DFT and time-dependent DFT (TD-DFT) as implemented in Gaussian^80^ and VeloxChem,^62^ respectively. The tensor-averaged damped cubic-response algorithm developed in this work was implemented in VeloxChem, whose source code is publicly available. Geometry optimizations employed B3LYP,^81,82^ functional with the 6–31G­(d,p)^83^ basis set, including Grimme’s D3­(BJ)^84^ dispersion correction to account for van der Waals interactions. Nonlinear optical response properties were computed with the MN15 ^85^ functional and the def2-SVPD^86^ basis using the damped cubic response formalism developed in this work. Molecular clusters were built from the experimentally obtained OTBP crystal structures reported by Du et al.^63^ to capture realistic intermolecular packing effects, with cluster sizes (*n* = 1, 2, 4, and 6) chosen to probe the evolution of THG behavior from isolated molecules to extended aggregates.

### 3.1 Resonance-Enhanced THG in OTBP: Multiphoton Mechanisms, Aggregation Effects, and Computational Scaling

#### 3.1.1 Multiphoton Resonances and Molecular Nonlinear Response of Isolated Chromophores

Du et al.^63^ experimentally demonstrated that OTBP exhibits markedly stronger third-harmonic generation (THG) than its structural analogue ODBP, attributing this enhancement to OTBP’s extended π-conjugation, reduced HOMO–LUMO gap, and increased transition dipole moment. These electronic-structure considerations provide valuable qualitative insight into the origin of OTBP’s superior THG response. To further complement their findings, we conducted a comparative computational analysis of the third-order hyperpolarizabilities of OTBP and ODBP to quantitatively elucidate the molecular mechanisms governing their differing THG efficiencies.

Figure 1 shows the frequency-dependent norm of the orientationally averaged third-order hyperpolarizability for OTBP and ODBP across the 550–1850 nm spectral range. Our calculations predict a pronounced THG resonance for OTBP at 1398 nm, in good qualitative agreement with the experimentally observed peak near 1300 nm. In contrast, ODBP displays a much weaker response. This confirms that the experimentally reported enhancement originates partially from intrinsic differences in their electronic structures and not just extrinsic effects such as aggregation.

**Figure 1:**
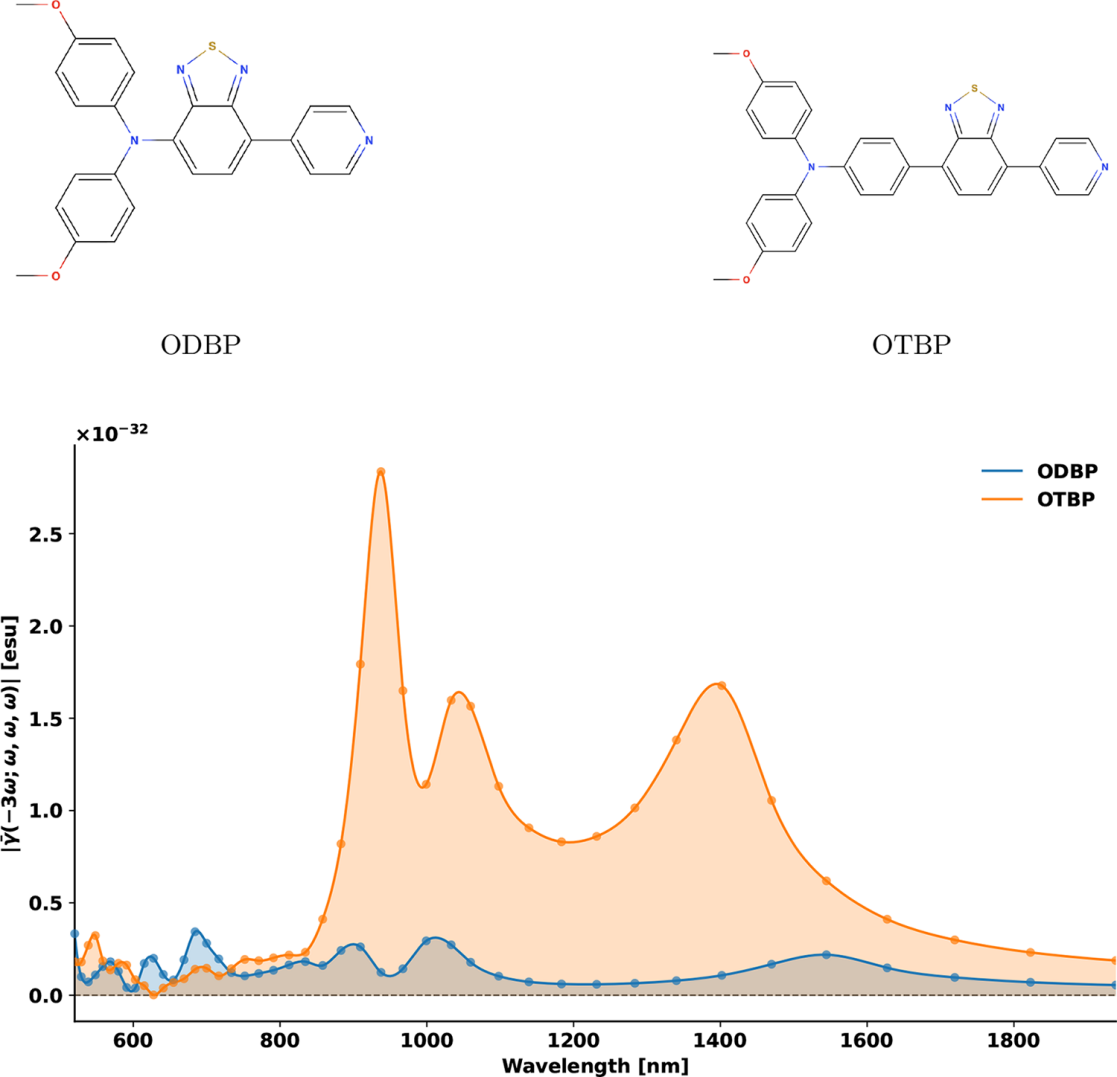
Molecular structures and frequency-dependent third-order hyperpolarizability. Chemical structures of ODBP and OTBP, highlighting the extended π-conjugation in OTBP. Frequency-dependent norm of the orientationally averaged third-order hyperpolarizability, |γ̅(−3ω; ω, ω, ω)|, for ODBP and OTBP. OTBP exhibits a pronounced resonance at 1398 nm, closely aligned with the experimental excitation window near 1300 nm, and demonstrates substantially enhanced response across the near-infrared spectrum compared to ODBP.

To further elucidate the electronic origins of THG resonance, we decomposed *γ̅* into its real and imaginary components, as shown in Figure 2. The imaginary part of γ for OTBP exhibits several distinct maxima that occur at the same spectral positions as the peaks in Figure 1. Because the imaginary component reflects dissipative energy flow through resonant transitions, these maxima indicate where resonance enhancement takes place in the THG response. However, while the imaginary component reflects resonance enhancement, it does not uniquely identify whether the resonance enhancement arises from one-, two-, or three-photon absorption processes. To resolve this ambiguity, we computed the relevant multiphoton transition strengths in Table. [Table tbl1].

**Figure 2:**
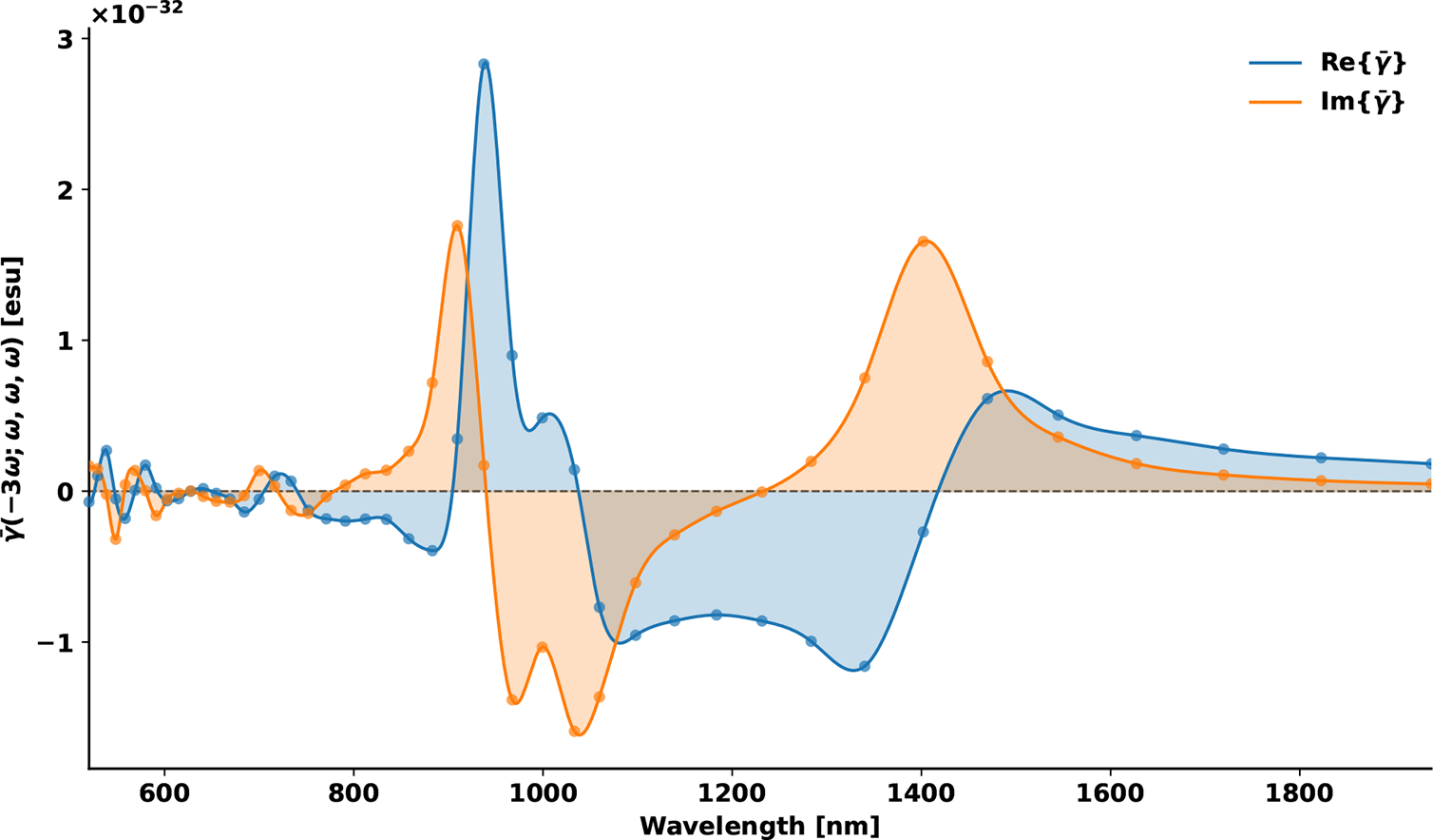
Complex components of the third-order hyperpolarizability. Real and imaginary components of γ̅(−3ω; ω, ω, ω) for OTBP. The enhanced imaginary features in OTBP within the 1300–1400 nm range confirm strong three-photon resonance at the experimental excitation wavelength, with the pronounced imaginary component indicating efficient resonant absorption processes.

**Table 1:** Calculated Two-Photon (2PA) and Three-Photon (3PA) Transition Properties for the First Five Excited States of OTBP^a^

	**wavelength (nm)**			**strengths**	
**transition**	**λ_1PA_**	**λ_2PA_**	**λ_3PA_**	**2PA** (×10^2^ a.u.)	**3PA** (×10^9^ a.u.)
S_0_ → S_1_	466	932	1398	659.97	15.91
S_0_ → S_2_	346	692	1039	476.03	66.58
S_0_ → S_3_	327	654	982	2.64	3.48
S_0_ → S_4_	312	624	936	14.59	3377.32
S_0_ → S_5_	288	577	866	8.23	0.14

a The column *λ*_1PA_ gives the one-photon resonance wavelength corresponding to the S_0_ → S_*n*_ energy gap. *λ*_2PA_ and *λ*_3PA_ denote the photon wavelengths required for two- and three-photon absorption, respectively, to excite the same transition. 2PA and 3PA strengths are given in atomic units.

The first peak in OTBP’s spectrum at 1398 nm corresponds to a three-photon resonance (3PA) associated with the S_0_ → S_1_ transition. Comparison with ODBP shows that OTBP’s 3PA strength for this transition (15.9 × 10^9^ vs 0.4 × 10^9^ a.u) is nearly 40 times larger. The second major peak at approximately 1039 nm also stems from a 3PA resonance, corresponding to the S_0_ → S_2_ transition. Finally, the strongest resonance for OTBP occurs near 930 nm, where two resonant processes coincide: a 2PA transition to S_0_ → S_1_ at 932 nm and a 3PA transition to S_0_ → S_4_ at 936 nm. This dual enhancement explains the pronounced increase in THG intensity in this spectral region.

Transition density maps (see Figure 3) further clarify the electronic factors underlying these observations. Both ODBP and OTBP exhibit charge transfer from the triphenylamine donor to the benzothiadiazole-pyridine acceptor in their S_0_ → S_1_ transition; however, OTBP shows a substantially greater degree of charge redistribution. The extended conjugated bridge in OTBP facilitates more extensive electron delocalization, producing a highly polarizable electronic environment conducive to strong third-order nonlinearity. This combined spectral and electronic analysis provides a molecular-level understanding of resonance enhancement in third-harmonic generation. Taken together, the spectral assignments and electronic-structure analysis offer a molecular-level understanding of the resonance enhancement mechanisms in THG. While the present study focuses on two specific chromophores, the computational framework developed here establishes a general route for linking structural features to their multiphoton resonance behavior. This capability enables systematic, a priori evaluation of candidate molecules and provides a basis for guiding the design of chromophores with tailored, wavelength-dependent nonlinear optical properties suitable for bioimaging and photonic applications.

**Figure 3:**
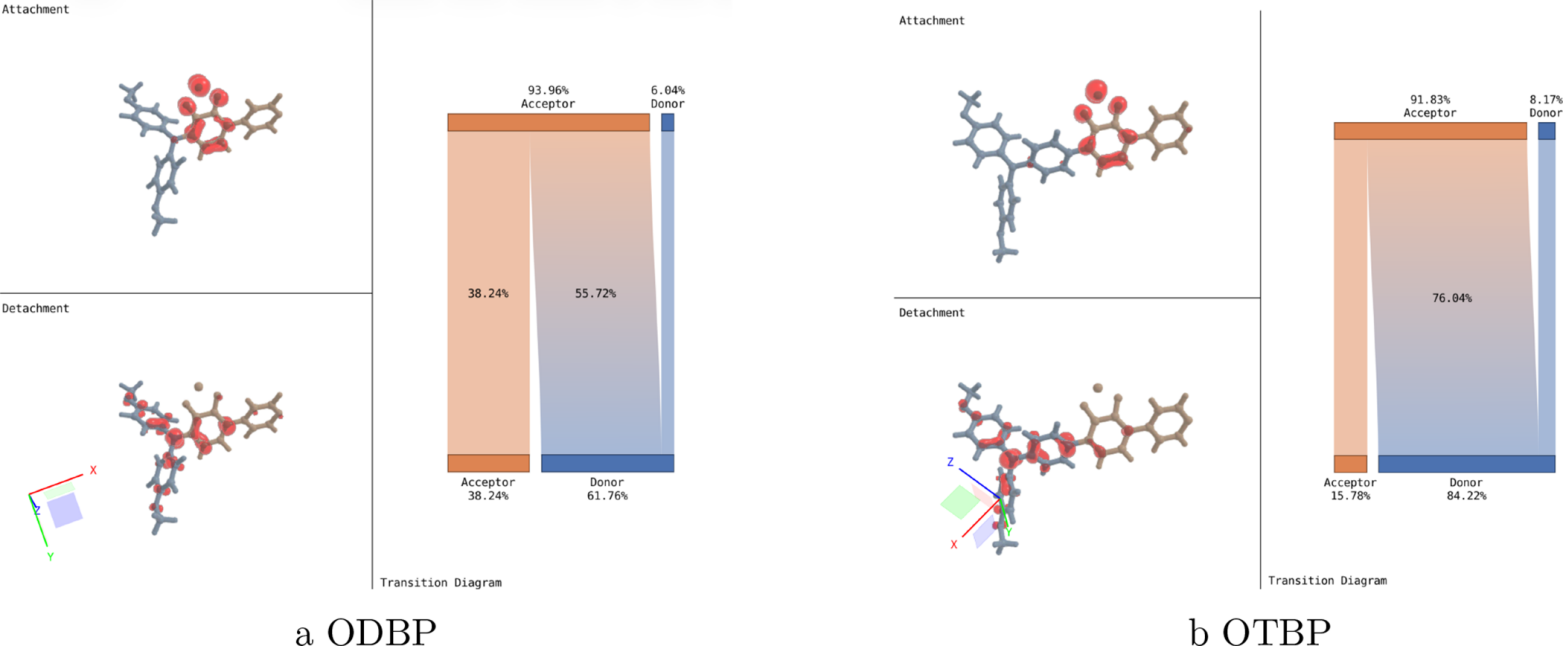
Transition density analysis. Attachment/detachment densities and transition-flow diagrams for (a) ODBP and (b) OTBP. The increased charge-transfer percentage in OTBP demonstrates enhanced donor–acceptor interaction, creating the highly polarizable electronic environment responsible for its superior third-order nonlinear response.

#### 3.1.2 Aggregation-Induced Modulation of Nonlinear Response

A more detailed picture of the aggregation-induced THG response emerges when the experimentally observed packing geometries of OTBP reported by Du et al. are examined.^63^ Four idealized intermolecular packing motifs, eclipsed, staggered, slipped, and tilted, can be identified as limiting cases of the relative orientations present in the crystal structure in Figure 5 and are summarized schematically in Figure 4. While these motifs are depicted as distinct in the schematic representation, real crystal dimers generally exhibit combinations of these geometric features, as illustrated by the representative structures shown in Figure 5. These motifs, therefore, provide a convenient geometric framework for rationalizing how electronic coupling is established between neighboring chromophores in the solid state.

**Figure 4:**
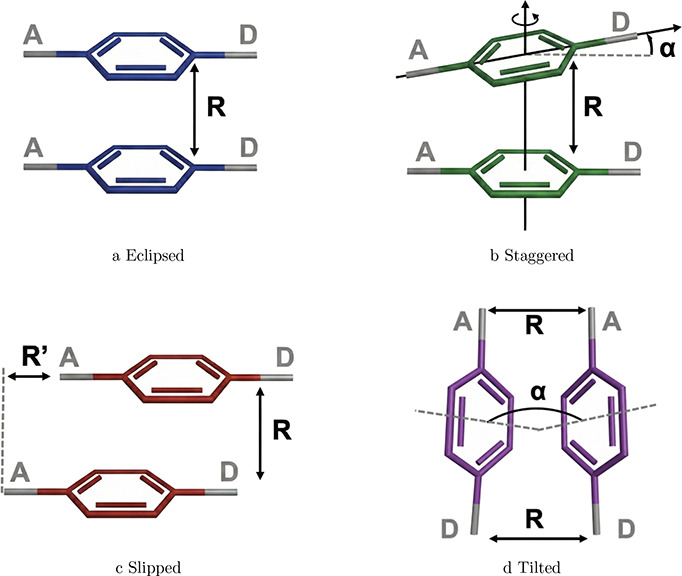
Schematic intermolecular packing motifs in OTBP. Illustration of the four distinct relative orientations observed between adjacent chromophores in the crystal structure: (a) eclipsed, (b) staggered, (c) slipped, and (d) tilted. These motifs define the geometric parameters—vertical separation *R*, lateral displacement *R′*, and torsional angle α—that govern the magnitude and sign of intermolecular electronic coupling in OTBP aggregates.

**Figure 5:**
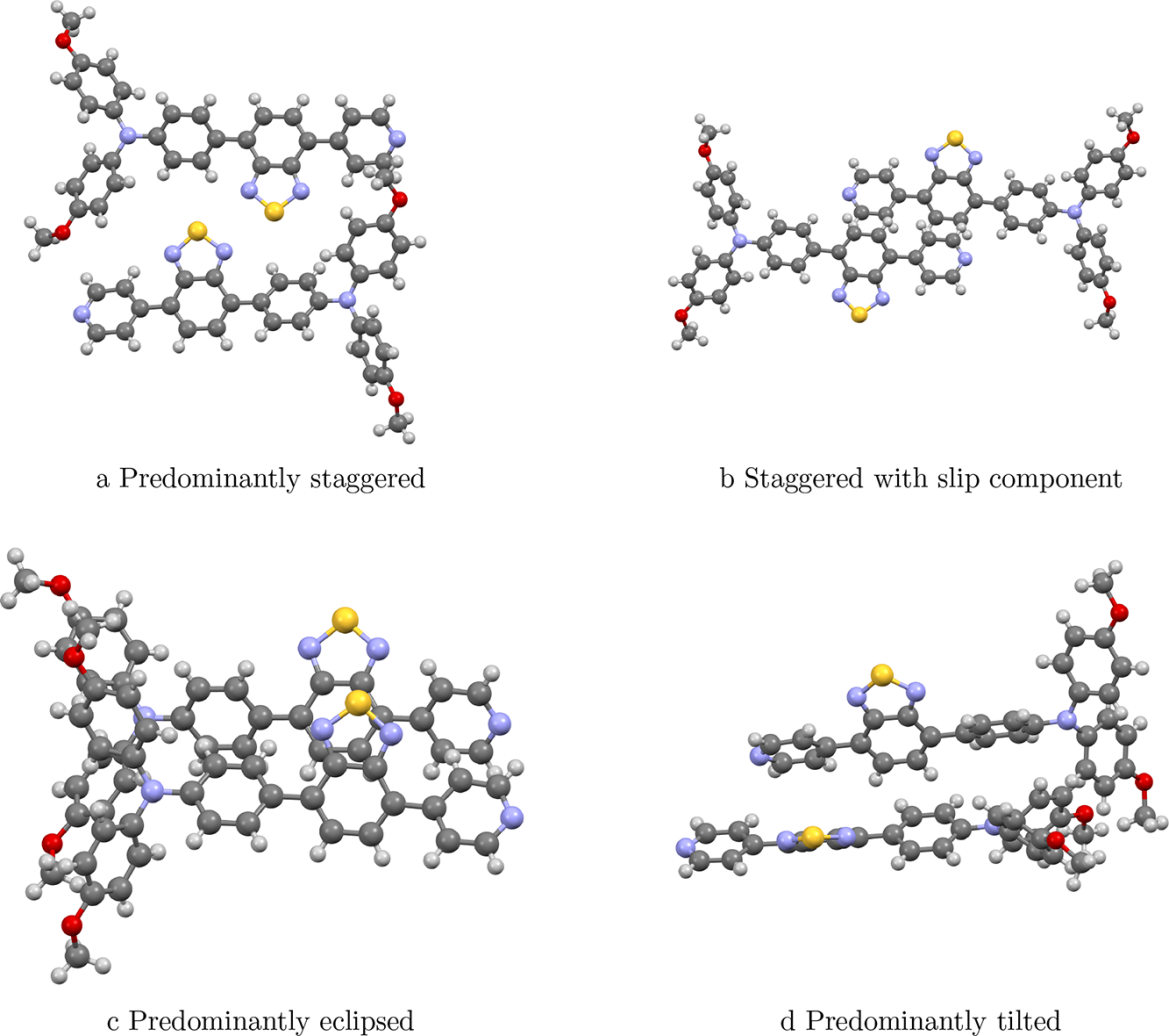
Intermolecular packing motifs of OTBP derived from the crystal structure. Representative dimer geometries extracted from the experimental unit cell. Each structure is categorized by its dominant geometric character relative to the idealized descriptors in Figure 4; in general, real dimers can simultaneously display more than one descriptor (e.g., staggered and slipped). Shown are (a) predominantly staggered, (b) staggered with a slip component (slipped-staggered), (c) predominantly eclipsed, and (d) predominantly tilted.

Figure 6 presents the frequency-dependent THG response for each representative packing geometry. Certain orientations are found to effectively quench nonlinear activity through destructive interference, whereas others promote cooperative enhancement. The horizontal reference lines indicate the maximum values of γ associated with the resonance enhancement at 1398 nm for one isolated chromophore and for two uncoupled monomers. The latter reference corresponds directly to the prediction of the oriented-gas model (eq 3), in which the third-order response is obtained by a simple additive sum of independent molecular contributions. The numerical factors annotated above each curve are normalized to the isolated-monomer response, enabling direct assessment of how the local packing environment modulates the effective third-order hyperpolarizability relative to the additive limit.

**Figure 6:**
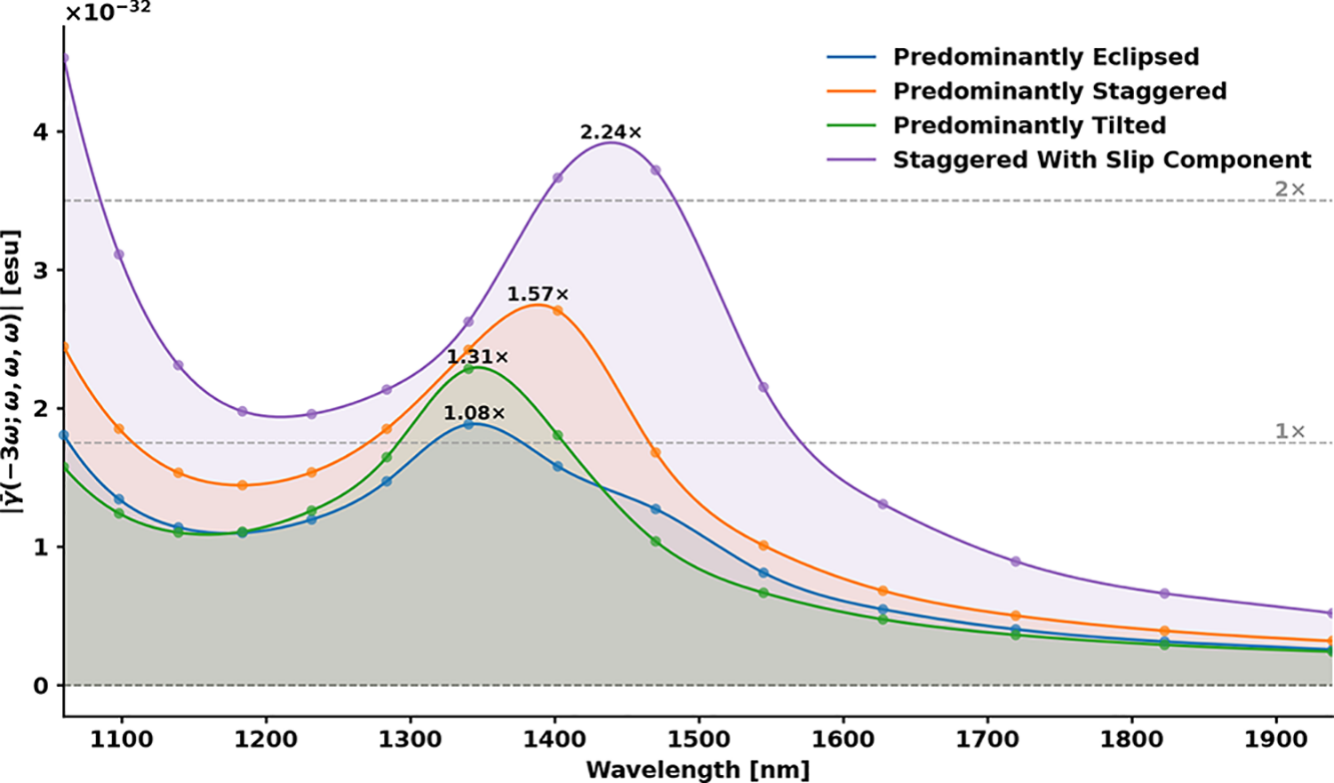
Third-harmonic generation response of distinct OTBP packing motifs. Calculated dispersion of the orientationally averaged third-order hyperpolarizability |γ̅(−3ω; ω, ω, ω)| for four intermolecular geometries observed in the crystal: eclipsed, staggered, slipped-staggered, and tilted. The dashed horizontal lines denote the THG amplitude of a single isolated OTBP molecule evaluated at its resonance maximum near 1398 nm, which corresponds to the dominant S_0_ → S_1_ resonance enhancement. The 1× line marks this isolated-monomer value, while the 2× line represents the response for two noninteracting monomers. Peak intensities of each packing motif are annotated relative to this isolated-monomer baseline, highlighting how specific intermolecular arrangements either suppress or enhance the resonant THG response.

The eclipsed geometry exhibits a peak intensity only slightly larger than that of a single monomer, indicating nearly complete cancellation of the contribution from the second chromophore and consistent with strong destructive interference between the induced dipoles. The staggered and tilted arrangements yield somewhat larger peak responses, but still remain well below the additive oriented-gas benchmark, demonstrating that these geometries also suppress cooperative enhancement. By contrast, the slipped-staggered motif produces clear amplification of the THG response, slightly exceeding the oriented-gas prediction for two noninteracting monomers. Together, these results reveal that both the sign and magnitude of intermolecular coupling in OTBP are highly sensitive to packing geometry.

The strong packing dependence found here is consistent with earlier theoretical work by Di Bella et al. on intermolecular effects in chromophoric assemblies, where a systematic analysis of dimer geometries showed that the nonlinear optical response depends sensitively on relative molecular orientation and can be enhanced in slipped donor–acceptor arrangements while being suppressed in less favorable packing motifs.^27^ Although that study focused on the first hyperpolarizability β and the second-order nonlinear response, the present results suggest that an analogous structural sensitivity also governs the orientationally averaged third-order response relevant to THG. In the present work, this analysis is included as an illustrative application of the tensor-averaged cubic-response formalism rather than as an exhaustive mapping of the multidimensional dimer packing landscape, which would be an interesting subject for a separate application-oriented study. This pronounced orientational dependence underscores the crucial role of crystal packing in governing macroscopic THG performance and highlights intermolecular alignment as a key design variable for optimizing organic nonlinear optical materials. Remarkably, the coupling between adjacent chromophores can reach magnitudes comparable to the hyperpolarizability of a single monomer, such that constructive or destructive interference can dominate the overall response. Such strong interactions lie well outside the regime of validity of the oriented-gas model and highlight the necessity of cluster-based computational approaches for capturing the true nonlinear optical behavior of molecular solids.

In Figure 7, the isotropic hyperpolarizability is plotted for an increasing number of monomers where the configuration used for *n* = 2 is the staggered and slipped configuration which displayed enhancement compared to two isolated monomers. Interestingly, a saturation is observed once going beyond *n* = 2.

**Figure 7:**
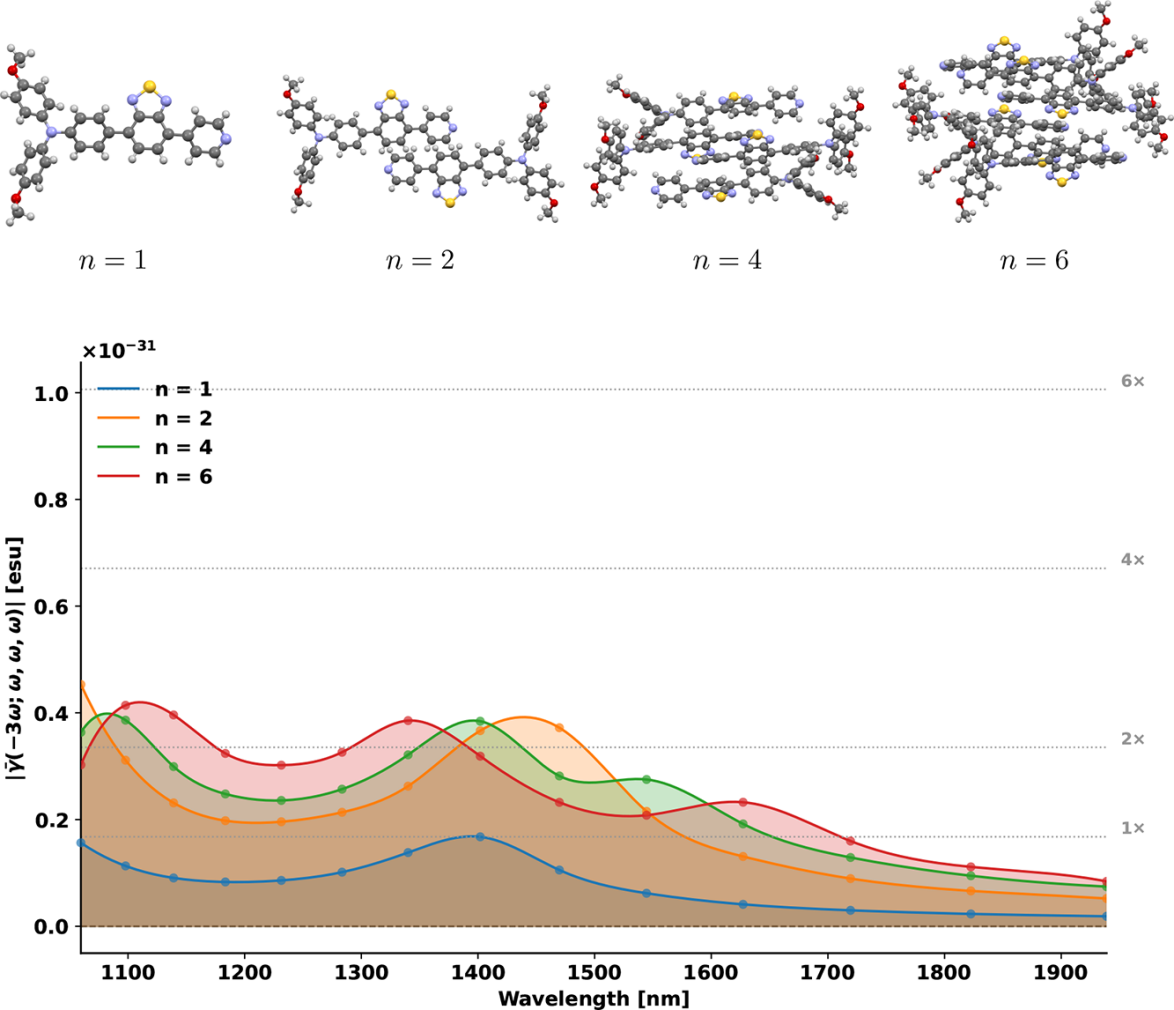
Third-harmonic generation response of OTBP clusters of increasing size. Calculated dispersion of the orientationally averaged third-order hyperpolarizability |γ̅(−3ω; ω, ω, ω)| for OTBP clusters containing *n* = 1, 2, 4, and 6 monomers, with all geometries taken directly from the experimental crystal structure. The dashed horizontal lines correspond to integer multiples (1×, 2×, 4×, and 6×) of the THG amplitude of a single isolated OTBP molecule evaluated at its resonance maximum near 1398 nm, associated with the dominant S_0_ → S_1_ transition. These reference levels illustrate how the nonlinear optical response saturates with increasing cluster size, reflecting the presence of both constructive and destructive intermolecular couplings within the crystal. Cubic response calculations were performed at the MN15/def2-SVPD level with a convergence threshold of 10^–3^ for the response vectors and evaluated at 15 excitation frequencies.

Our computational results show that crystal packing has a very large effect on the resulting hyperpolarizability and that crystallization design is imperative for the development of new nonlinear optical materials. We believe that the developed computational framework enables the rational design of novel NLO materials, including the effect of crystal packing.

#### 3.1.3 Computational Efficiency and Scaling of Tensor-Averaged Cubic Response

To assess both the physical scaling behavior and the computational efficiency of the tensor-averaged cubic-response formulation, we consider OTBP in its monomeric form as well as molecular clusters with sizes *n* = 2, 4, and 6 constructed directly from the experimental crystal structure. The resulting system sizes, reaching up to 372 atoms, more than 1600 electrons, and nearly 5800 contracted basis functions for the hexamer, are summarized in Table 2.

**Table 2:** System Size of OTBP Monomers and Molecular Aggregates Used in the Present Work^a^

system	atoms	electrons	contracted basis functions
monomer	62	270	965
dimer	124	540	1930
tetramer	248	1080	3860
hexamer	372	1620	5790

a Each OTBP aggregate was constructed directly from the experimental crystal structure and treated by using the damped cubic response formalism. The table highlights the rapid growth in system size, reaching several hundred atoms, over 1600 electrons, and roughly 5800 contracted basis functions for the hexamer.

For each system, the isotropically averaged second hyperpolarizability γ̅(−3ω; ω, ω, ω) was evaluated at 15 excitation frequencies using the damped cubic-response formalism and averaged according to eq 5, yielding the THG spectra shown in Figure 7. Modern response implementations already offer two decisive advantages for such multifrequency nonlinear calculations. First, the use of lightweight two-electron integral engines with minimal memory footprint allows many auxiliary Fock matrices to be constructed efficiently in parallel from a single batched evaluation of electron repulsion integrals.^62^ Second, reduced-space response solvers permit the simultaneous treatment of multiple excitation frequencies within a common iterative subspace, such that entire spectral windows can be converged at essentially the cost of a single frequency.^53,54^ . However, even within this already optimized framework, the explicit evaluation of all Cartesian tensor components of the cubic response remains a major computational bottleneck. Each tensor element γ_αβγδ_ requires independent cubic and quartic electronic-Hessian contractions, and each contraction necessitates the construction of multiple transformed Fock matrices. Although the underlying two-electron integrals can, in principle, be evaluated only once, the use of large basis sets implies that the full set of auxiliary Fock matrices cannot be constructed and stored simultaneously. As a result, the Fock matrices must be generated in successive memory-limited batches. Furthermore, the repeated linear transformations required to form these auxiliary Fock matrices within each batch incur substantial computational and memory overhead. This overhead increases rapidly with system size and becomes the dominant cost in large-scale third-harmonic generation calculations. This bottleneck is entirely associated with the requirement for several spatial components of the response tensor. In the conventional tensor-component formulation, the contraction of the quartic Hessian in eq 31 alone requires 66 distinct Fock matrix constructions per frequency, 6 from the first-order contributions in eq 35, 18 from the second-order terms in eq 36, and 42 from the third-order terms in eq 37. Similarly, the cubic-Hessian contraction in eq 34 requires an additional 60 Fock constructions per frequency. In total, the conventional Cartesian approach therefore requires 126 unique Fock matrix builds per frequency. These Fock matrices must be explicitly constructed and stored, introducing substantial computational and memory costs that ultimately limit scalability.

By contrast, the tensor-averaged formulation introduced in this work exploits the linearity of the Fock matrix construction and the subspace-extraction method. By performing the orientational averaging analytically at the level of the perturbed densities and transformed Fock matrices, all redundant Cartesian tensor components are eliminated before the electronic-Hessian contractions are evaluated. As a result, only 24 Fock matrix constructions per frequency are required corresponding to a reduction of 72.7% for the quartic-Hessian contraction and 90.0% for the cubic-Hessian contraction, as summarized in Table 3. Overall, this amounts to a more than 6-fold reduction in the total number of Fock builds per frequency. This reduction is complementary to the existing frequency-domain optimizations: while multifrequency subspace solvers minimize the number of iterations required across the spectral window, the tensor-averaged formulation minimizes the number of Cartesian-related computations that must be evaluated. In this sense, the present method constitutes a spatial-domain optimization that operates on top of already highly efficient integral-driven and frequency-resolved response infrastructure.

**Table 3:** Number of Fock Matrices per Frequency for the Calculation of the Orientational Average Hyperpolarizability of ([Disp-formula eq5]) Using the Tensor-Component and Tensor-Averaged Algorithms^a^

	tensor component	tensor average
first-order		
*f*_α;σ_^ω^	6	0
*f*_α,β;σ_^ω,ω^	18	12
*f*_α,β,γ;σ_^ω,ω,ω^	42	6
second-order		
*f*_α;σ_^2ω^	6	0
*f*_α,β;σ_^ω,2ω^	54	6
total Fock matrices	126	24

a The “Tensor component” column refers to the conventional cubic-response formulation in which all Cartesian tensor elements γ_αβγδ_ are evaluated explicitly via the cubic- and quartic-Hessian contractions of eqs 32 and 31. The “Tensor average” column corresponds to the isotropic formulation introduced in this work, where the orientational average is applied at the level of the perturbed densities and transformed Fock matrices through the compounded contractions of eqs 50 and 51, eliminating redundant exchange–correlation kernel integrations while fully preserving the exact isotropic cubic response.

As the construction of Fock matrices overwhelmingly dominates the computational cost, the savings made in the number of matrices closely relate to reduced (start-to-end) wall times in the THG simulations and, in turn, enabling the treatment of systems of the sizes addressed in the present work with use of today’s standard cluster resource allocations. As GPU-accelerated techniques are actively being developed at the moment,^87^ any form of wall time reports are bound to quickly become outdated. But just to give an idea of things, we note that the entire spectrum calculation or, in other words, the tensor-averaged quantity γ̅(−3ω; ω, ω, ω) for the set of 15 frequencies for the monomer in Figure 7 took 3.3 h with use of a single dual-socket node equipped with AMD EPYC Zen2 64-core CPUs. To put this computational effort in a relative perspective, the corresponding calculation of a single tensor component, γ_*xxzz*_(−3ω; ω, ω, ω), took 1.5 h using the same amount of resources. We can thus conclude that our work makes THG spectrum calculations viable whenever the calculation of single γ-tensor components can be afforded.

Figure 8 explicitly illustrates the distribution of Fock matrix builds across different stages of the THG calculation. The first two categories correspond to the iterative solution of the linear and quadratic response equations for the wave function parameters, while the last two categories represent the electronic-Hessian tensor contractions required for the cubic response.

**Figure 8:**
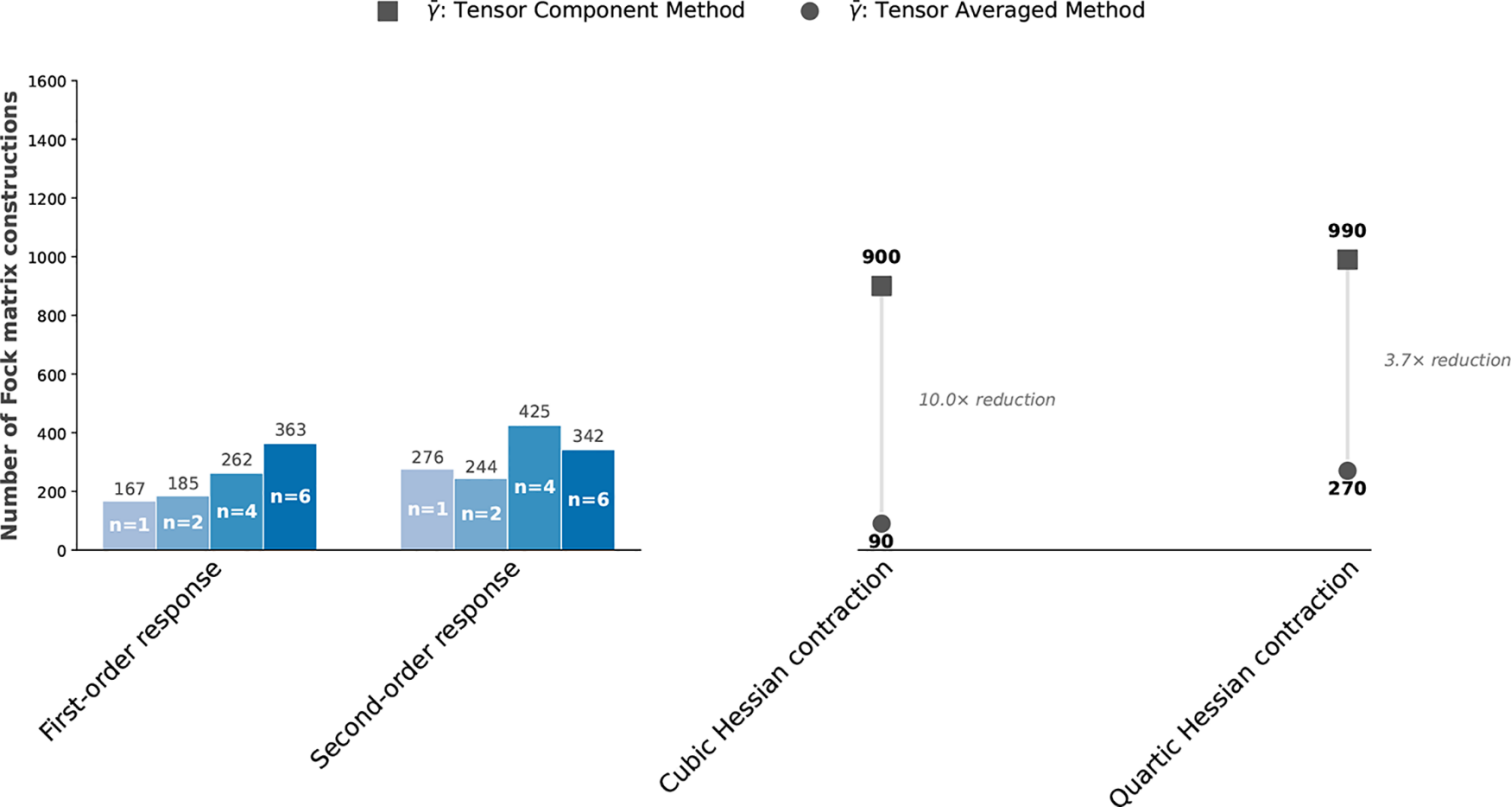
Number of Fock matrix constructions required for each stage of the third-harmonic generation (THG) calculation. The first two categories correspond to the evaluation of the first- and second-order response amplitudes, $\frac{d\kappa^{\omega_{1}}}{dF_{\beta }^{\omega_{1}}}$ and $\frac{d^{2}\kappa^{\omega_{2},\omega_{3}}}{dF_{\beta }^{\omega_{2}}dF_{\gamma }^{\omega_{3}}}$ , obtained from the linear and quadratic response equations, eqs 26 and 27, respectively, and shown for systems with *n* = 1, 2, 4, and 6 units. The last two categories represent the electronic-Hessian tensor-contraction steps entering the cubic response function: the conventional tensor-component cubic and quartic-Hessian contractions, eqs 31 and 32, and their corresponding tensor-averaged counterparts introduced in this work, eqs 50 and 51, which directly yield the isotropic cubic response γ̅.

The number of Fock matrix constructions associated with the first- and second-order response amplitudes does not depend on system size, but is governed by the convergence behavior of the iterative subspace solver. In the employed symmetrized subspace procedure, each accepted trial vector gives rise to a Hessian-vector transformation and hence to the construction of an auxiliary transformed Fock matrix. Consequently, the number of such Fock builds is determined by the size of the reduced response space required to reach the target residual threshold. This subspace size is influenced by several factors, including the quality of the preconditioner, the proximity of the chosen frequency to resonance, the density of states in the relevant spectral region, and the degree of linear dependence among trial vectors removed during orthogonalization. Moreover, when several frequencies are treated simultaneously, linear dependence between trial vectors associated with different frequencies may reduce the number of distinct Hessian transformations that must be carried out. When the tensor-averaged algorithm is employed, the computational effort associated with the electronic-Hessian contractions is drastically reduced, and the dominant cost is shifted to the solution of the linear and quadratic response equations themselves. This redistribution of computational workload confirms that the present tensor-averaged formulation effectively removes the principal spatial bottleneck of the cubic-response theory while preserving the full accuracy of the isotropic THG response.

## 4 Conclusions and Outlook

We have developed an efficient and scalable cubic-response framework within time-dependent Kohn–Sham density functional theory for the simulation of frequency-dependent third-harmonic generation in extended molecular systems. Central to this development is an analytic tensor-averaged formulation that constructs isotropic third-order response quantities directly at the level of the electronic-structure equations. By eliminating the explicit construction of the full rank-four hyperpolarizability tensor, the present approach reduces the computational cost of THG calculations by nearly an order of magnitude and removes a long-standing spatial bottleneck in the cubic response theory.

The methodology was applied to oligo­(thiophene-benzothiadiazole) molecular aggregates to elucidate how resonance enhancement and crystal packing jointly govern the THG response in realistic condensed-phase environments. While the OTBP chromophore exhibits a large intrinsic molecular hyperpolarizability and strong resonance enhancement in the near-infrared region, explicit treatment of the experimental crystal packing reveals that aggregation does not universally enhance the electronic contribution to the third-order response. Instead, the dominant packing motifs give rise to damped cooperative effects. Slipped intralayer donor–acceptor dimers display constructive coupling and superlinear enhancement of γ­(−3ω; ω, ω, ω), whereas the more abundant interlayer motifs exhibit net suppression of the third-order response relative to infinitely separated chromophores.

These results demonstrate that the high THG efficiency of OTBP-based nanocrystals observed experimentally arises from a delicate interplay between large intrinsic molecular nonlinearity and morphology-dependent electromagnetic field enhancement, while the specific crystal packing leads to a partial suppression of the underlying electronic contribution to χ^(3)^. More broadly, the present framework provides a quantitative first-principles tool for disentangling intrinsic molecular effects from supramolecular and morphological contributions, and for assessing the potential for further performance gains through crystal-structure engineering. In this way, theory can directly guide experimental efforts by identifying packing motifs that maximize cooperative enhancement and those that are detrimental, enabling rational optimization of nonlinear optical materials at the structural level.

From a computational perspective, the tensor-averaged formulation shifts the dominant cost in large-scale calculations from Fock matrix construction to the solution of the linear and quadratic response equations. This identifies clear directions for future algorithmic development, including improved multifrequency response solvers and reduced scaling linear algebra strategies. The present framework is readily extendable beyond THG to other third-order nonlinear processes, such as four-wave mixing and the optical Kerr effect. Taken together, these advances establish a practical route toward material-level modeling and the rational design of nonlinear optical systems based on an accurate electronic-structure theory.

## Data Availability

The data that support the findings of this study are available from the corresponding author upon reasonable request.
